# Supplementary material for: SURF: integrative analysis of a compendium of RNA-seq and CLIP-seq datasets highlights complex governing of alternative transcriptional regulation by RNA-binding proteins
Source: Genome Biol. 2020 Jun 12;21:139. doi: 10.1186/s13059-020-02039-7 (PMC7291511; doi:10.1186/s13059-020-02039-7)
Supplement: Supplementary file 1 — Additional file 1 Additional file 1 contains supplementary Figs. S1-S44 and Tables S1-S7. [file 13059_2020_2039_MOESM1_ESM.pdf]

Supporting Information

Fan Chen[1] and Sündüz Keleş*[1,2]

[1]Department of Statistics,
[2]Department of Biostatistics and Medical Informatics,
University of Wisconsin–Madison

The document provides supporting information to "SURF: integrative analysis of a compendium of RNA-seq and CLIP-seq datasets highlights complex governing of alternative transcriptional regulation by RNA-binding proteins." This contains supplementary Figs. S1-S44 and Tables S1-S7.

*Correspondence: keles@stat.wisc.edu

Supplementary Figures

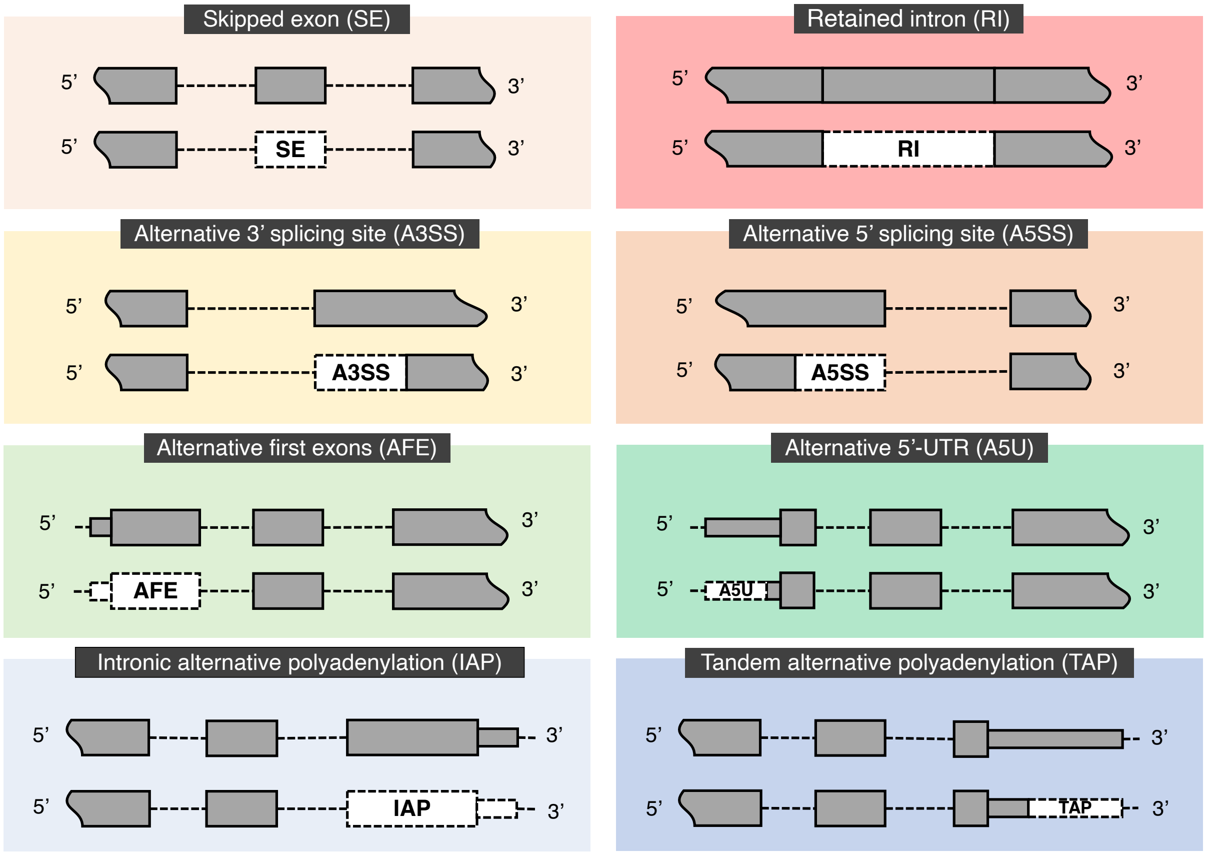

Fig. S1: Illustration of eight ATR event types: exon skipping (SE), alternative 3′ (A3SS) and 5′ (A5SS) splicing, and intron retention (RI) within the AS class; alternative first exon (AFE) and alternative 5′UTR (A5U) within the ATI class; intronic (IAP) and tandem (TAP) alternative polyadenylation within the APA class. In each panel, the upper track depicts part of a gene model, and the lower track demarcates a specific ATR event in a transcript (isoform) with a white dashed box.

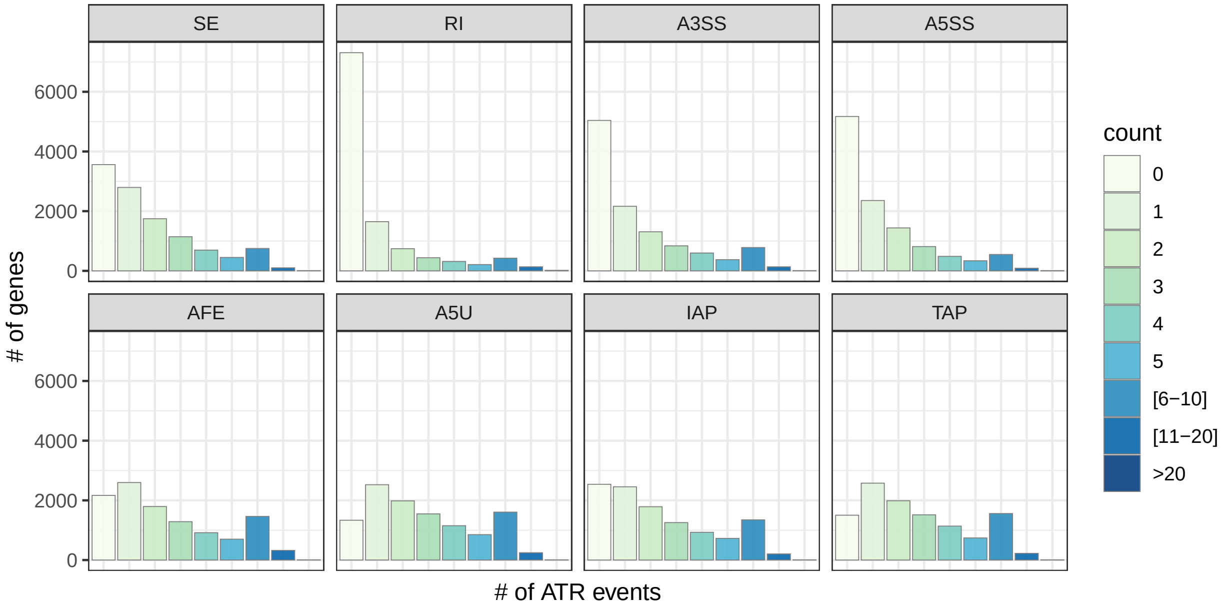

Fig. S2: Distribution of numbers of ATR events parsed out from human genome annotation (GENCODE version 24), stratified by the event type.

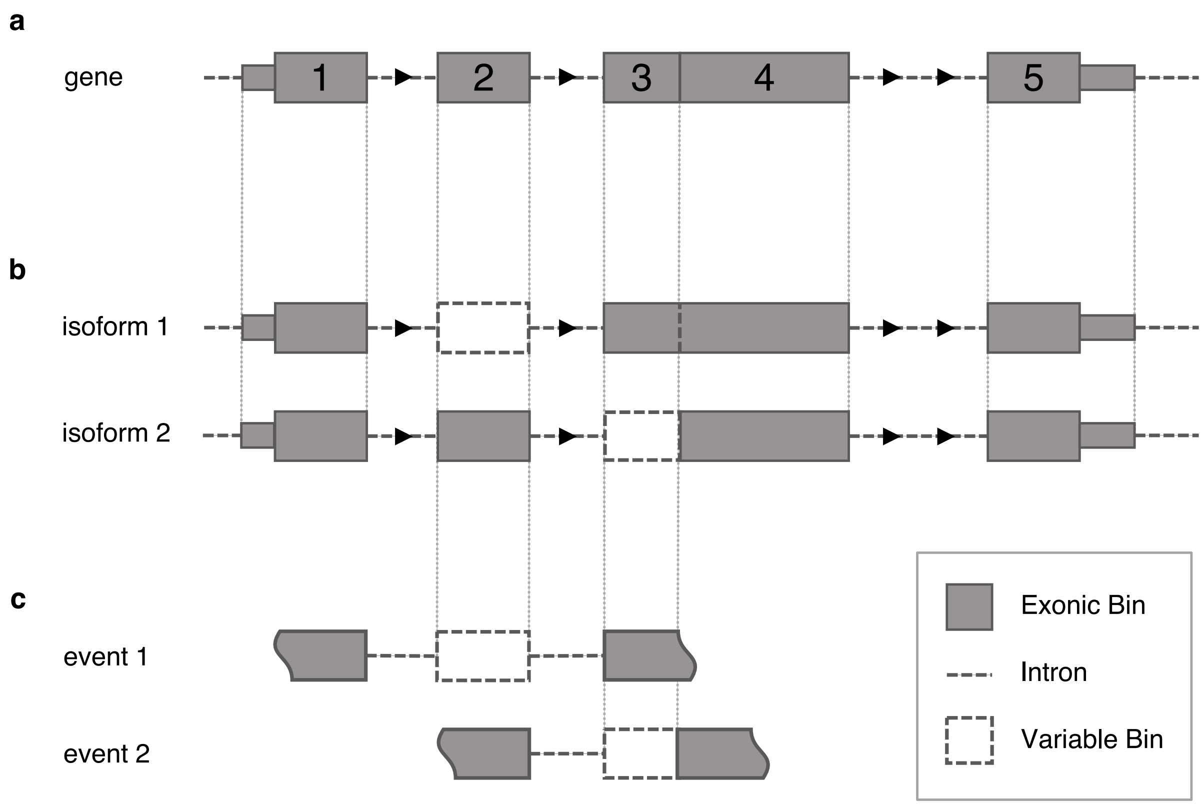

Fig. S3: Identification of ATR events from genome annotation. (a) All exons of the same gene are discretized into mutually disjoint exonic bins (numbered grey boxes). (b) Then, for every transcript (isoform) from the same gene, absent exonic bins are labeled as variable bins (white dashed boxes, e.g., exonic bin # 2 is a variable bin for isoform 1 and exonic bin # 3 is a variable bin for isoform 3). (c) Finally, within each transcript, consecutive variable bins are merged together and are labeled as an ATR event. A transcript can harbor multiple ATR events.

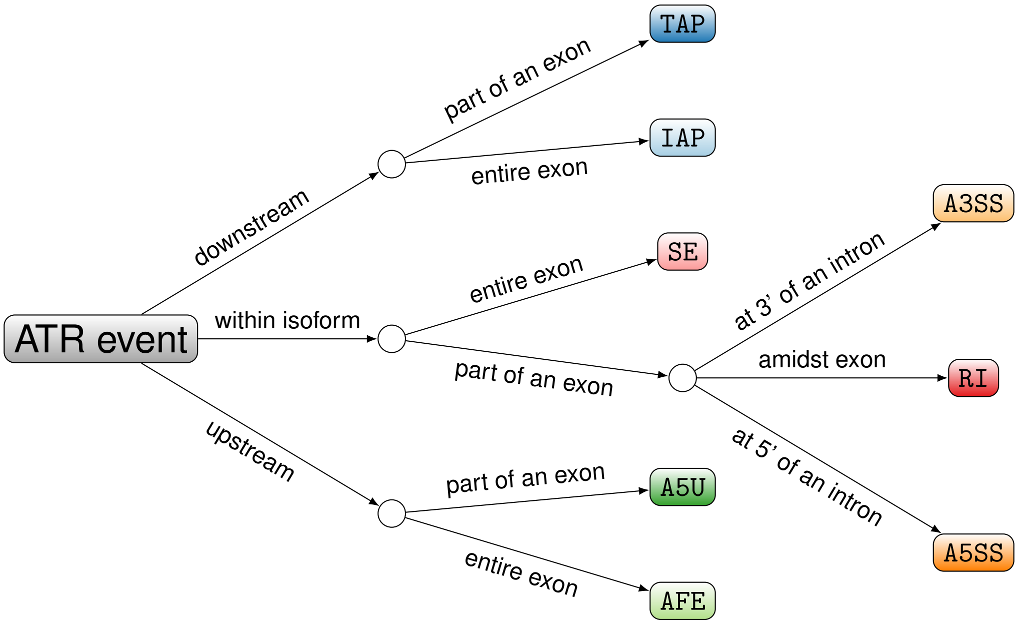

Fig. S4: Decision tree for annotating variable bins as ATR events. The decision tree takes a run of variable bins as input and classifies it into one of the eight event types. "downstream" and "upstream" refer to whether the variable bin is at the 3′ or 5′ end, respectively.

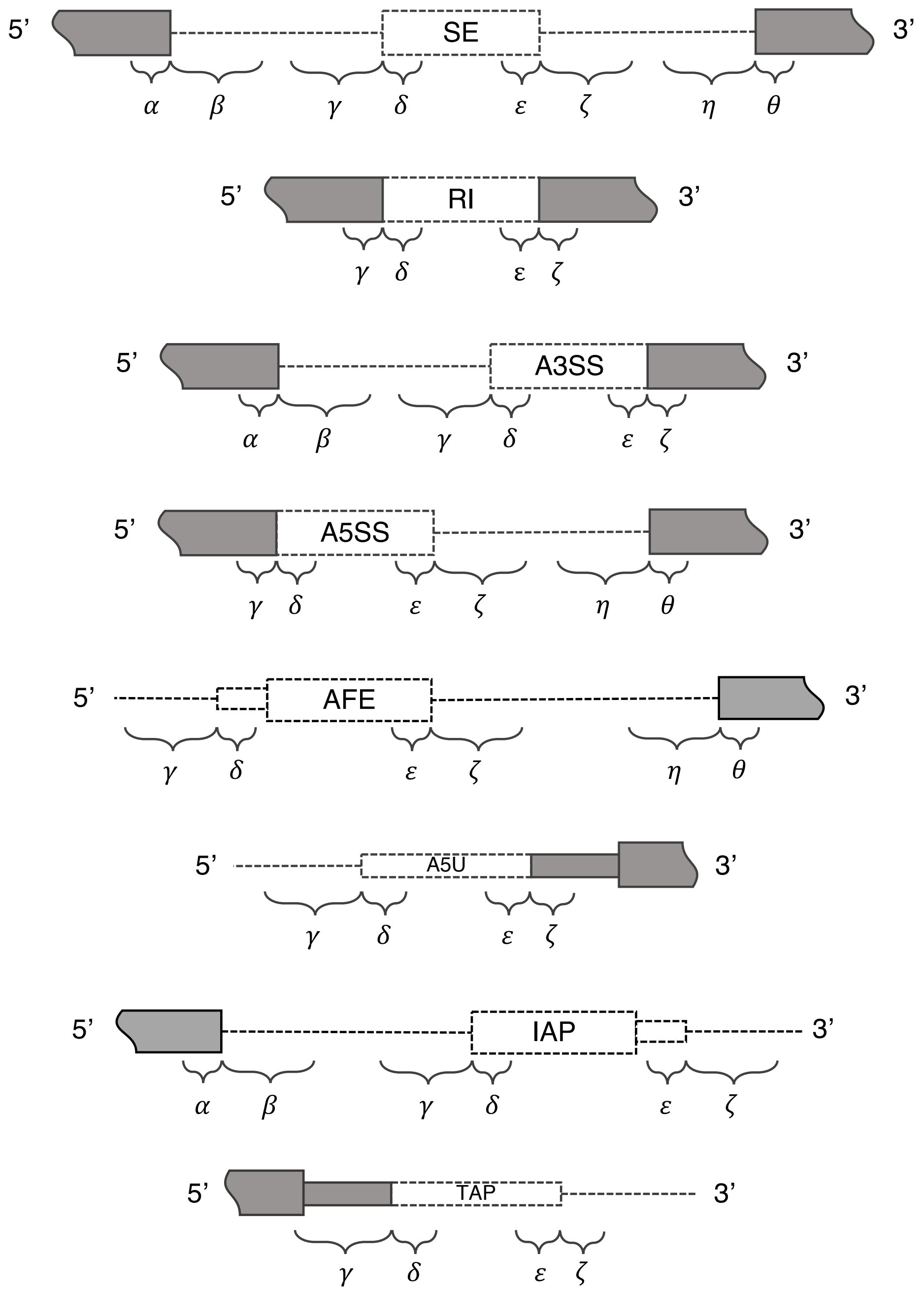

Fig. S5: Illustration of location features for eight ATR event types. White boxes depict the ATR events with the event type labeled inside. Short and long curly brackets correspond to genomic regions of length 100bp and 300bp respectively.

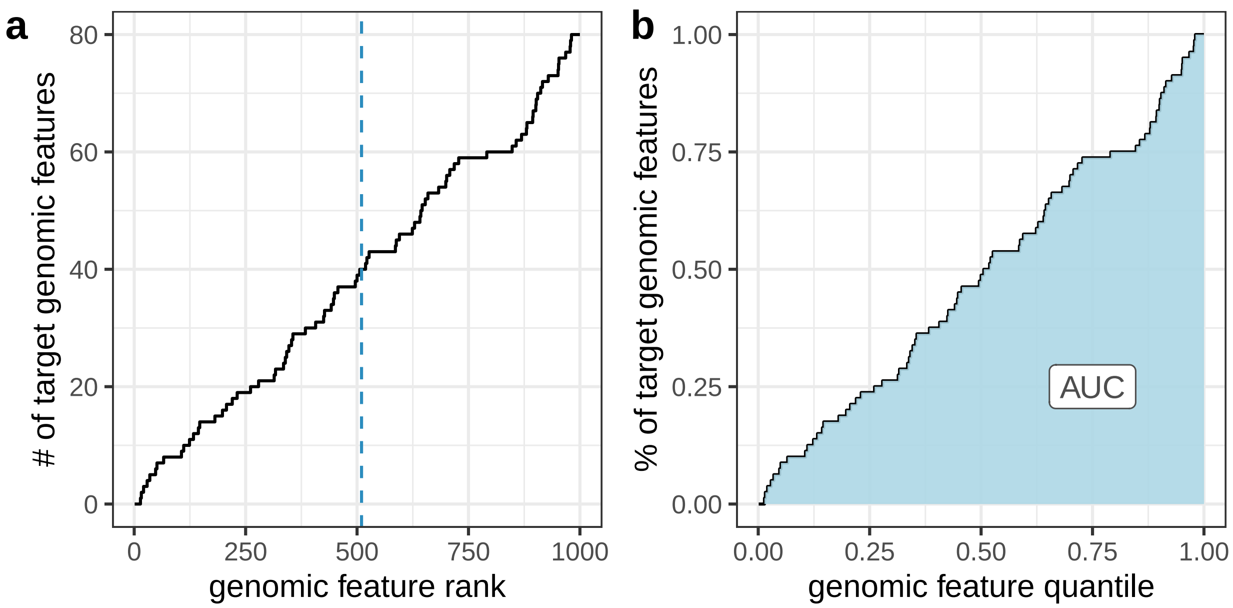

Fig. S6: Illustration of AUC calculation in discovery module of SURF. (a) Given a target genomic feature set (e.g., gene set or transcript set) and a descending ordering of all the features (e.g., ranked genes based on gene expression), the recovery curve (solid line) depicts the number of target genomic features among the top-ranked genomic features. For example, the set of top 510 features (marked by the vertical blue dashed line) harbors 40 target features. (b) Recovery curve in percentage scale. Both axes are re-scaled into $[0, 1]$. The blue area under the recovery curve (AUC) quantifies the activity level of target genomic set.

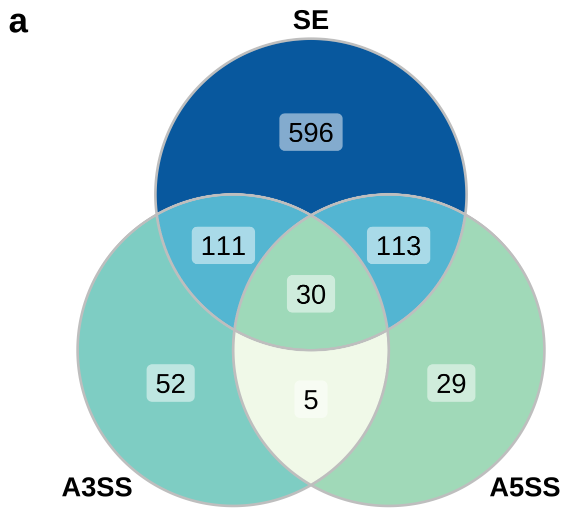

Fig. S7: Annotation of local splice variations (LSV) detected by MAJIQ on simulated RNA-seq data (Materials and Methods) into ATR event types. MAJIQ annotates most LSVs into three ATR event types: SE, A3SS, and A5SS; however, mappings from LSVs to ATR event types are not unique. (a) ATR event type annotations of 936 (out of 974) LSVs detected with the default settings of MAJIQ. (b) ATR event type annotations of 2237 (out of 2323) LSVs detected with a liberal configuration of MAJIQ, where the change threshold is set to 0.1 (via option `--threshold`).

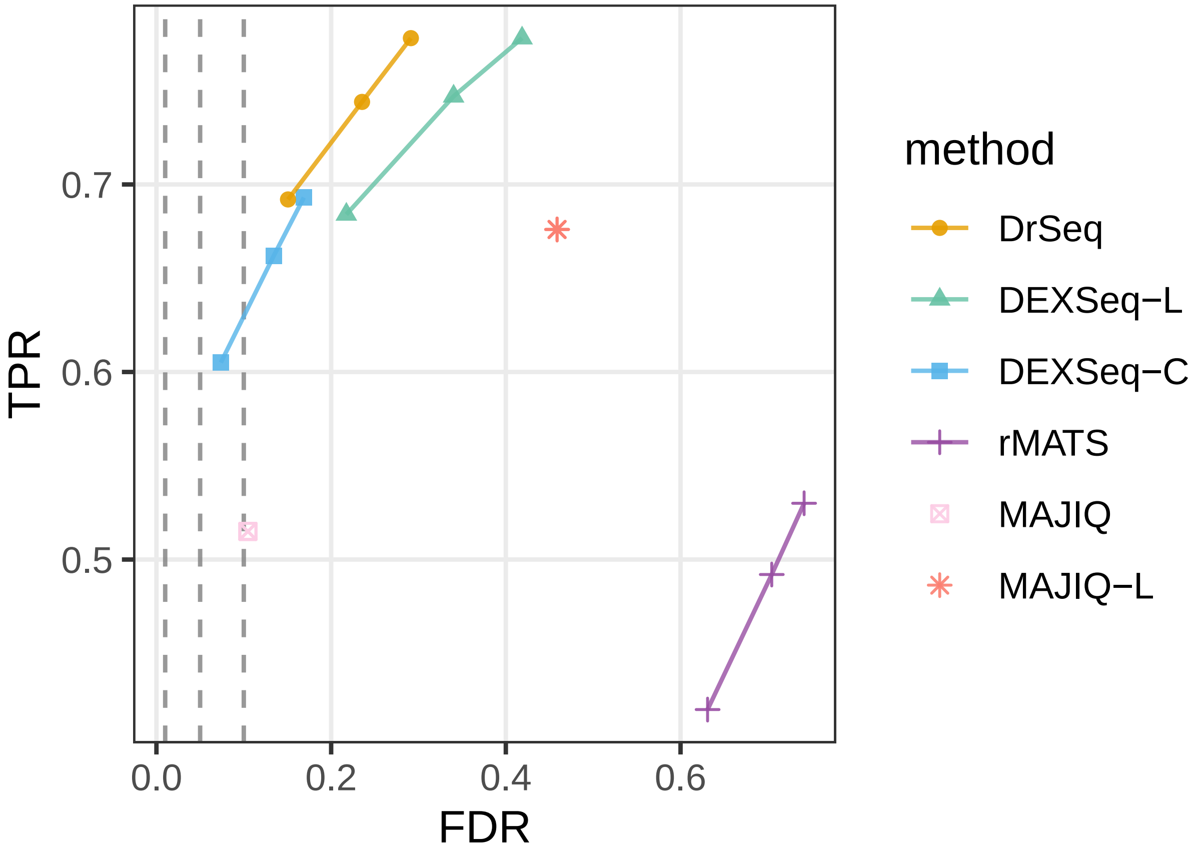

Fig. S8: Comparison of the aggregate performances of differential ATR event detection methods DrSeq, rMATS, two simple strategies for stitching DEXSeq inferences into ATR level (DEXSeq-L and DEXSeq-C), MAJIQ (default), and MAJIQ-L (liberal as described in Fig. S7) using simulated RNA-seq data. For the first four methods, the three points display the true positive rate (TPR) and observed false discovery rate (FDR) at target FDR levels of 0.01, 0.05, and 0.1, respectively. The MAJIQ variants do not report (adjusted) p-values for the tested local splice variations, thus their performances are summarized as single points.

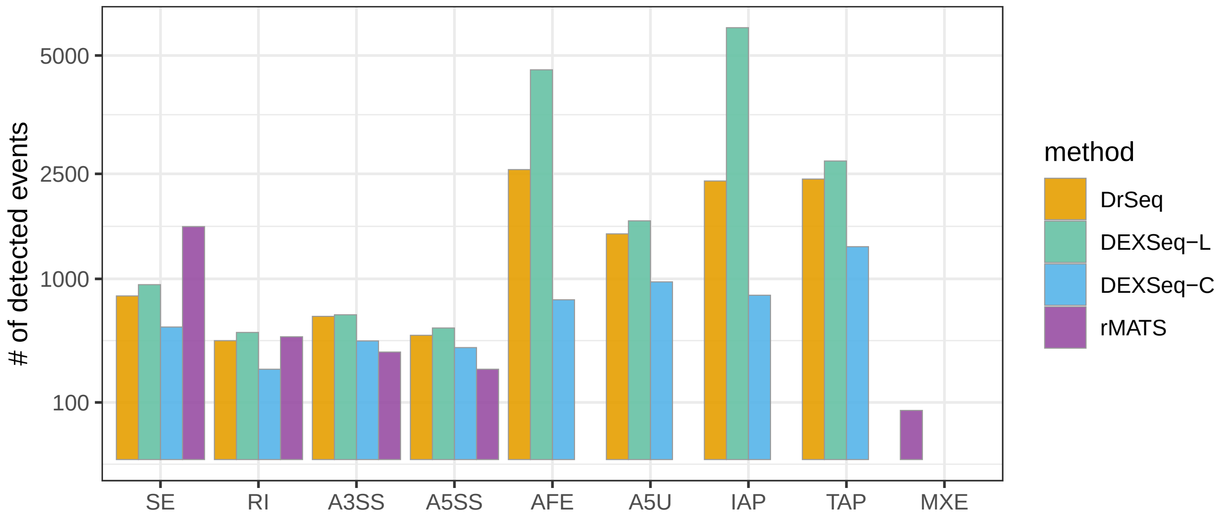

Fig. S9: Comparison of the number of detected differential ATR events by various event-based methods in human transcriptome-based simulations. rMATS does not identify ATR event types AFE, A5U, IAP or TAP. In contrast, the only category excluded from DrSeq's ATR repertoire is MXE (mutually exclusive exons).

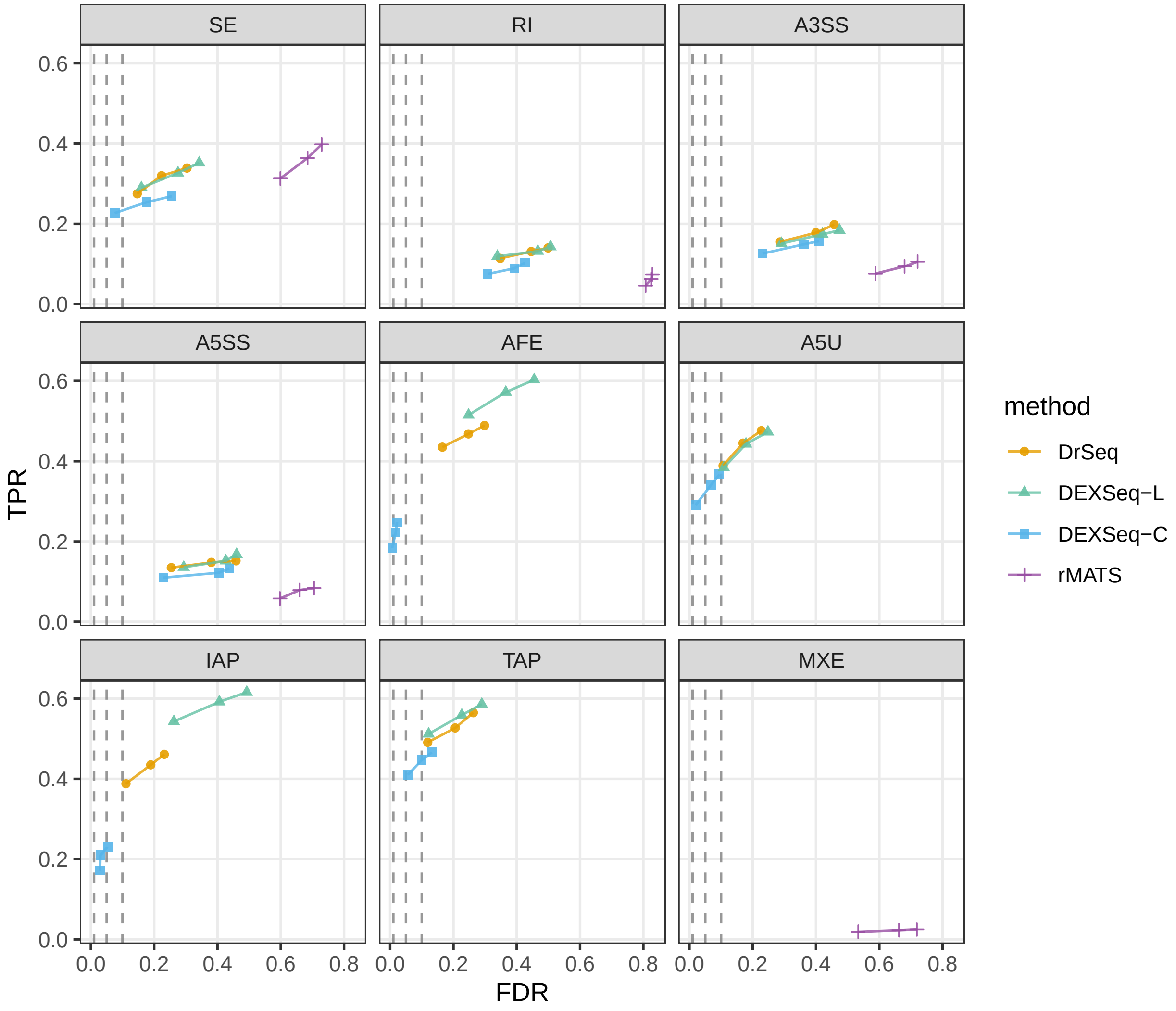

Fig. S10: Event type specific comparison of the performances of DrSeq, rMATS, and two simple strategies for stitching DEXSeq inferences into ATR event level (DEXSeq-L and DEXSeq-C) using simulated RNA-seq data. For each method, the three points display the true positive rate (TPR) and observed false discovery rate (FDR) at target FDR levels of 0.01, 0.05, and 0.1, respectively. rMATS does not identify ATR event types AFE, A5U, IAP or TAP. In contrast, the only category excluded from DrSeq's ATR event construction is MXE (mutually exclusive exons).

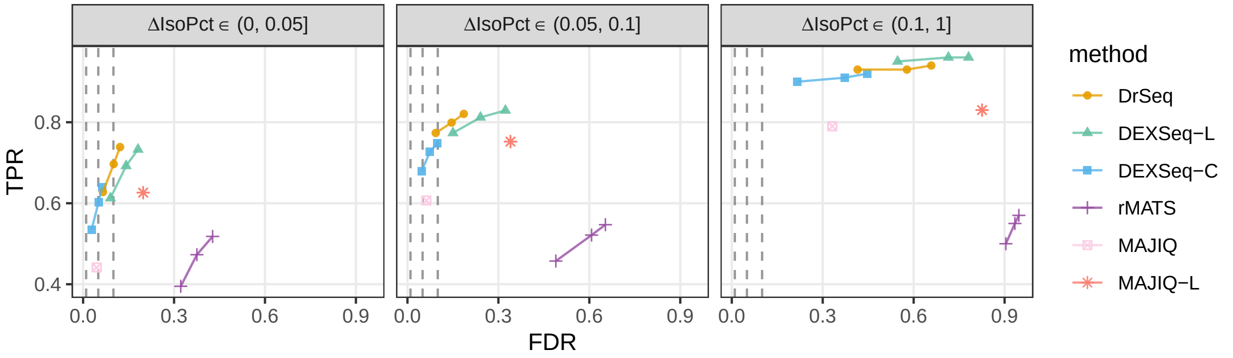

Fig. S11: Comparison of the performances of DrSeq, rMATS, MAJIQ, and two simple strategies for stitching DEXSeq inferences into ATR event level (DEXSeq-L and DEXSeq-C) under different signal-to-noise levels. All 16,824 genes with multiple isoforms are divided into three groups by the difference in their isoform percentages (ΔIsoPct) of the most expressed isoforms (in TPM), namely $(0, 0.05]$, $(0.05, 0.1]$, and $(0.1, 1]$. The number of genes with altered isoform expression (Materials and Methods) in three groups are 666, 234, and 100, respectively. For each method, the three points display the true positive rate (TPR) and observed false discovery rate (FDR) at target FDR levels of 0.01, 0.05, and 0.1, respectively. Two MAJIQ variants do not directly report (adjusted) p-values for the tested local splice variations, thus their performance are each depicted as a single point.

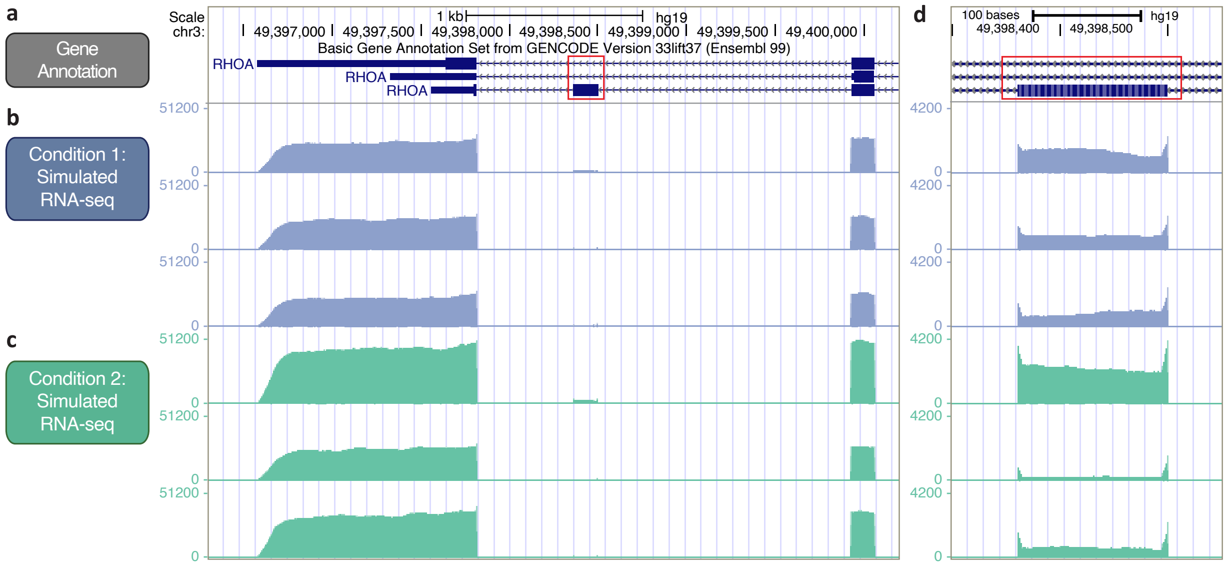

Fig. S12: A false positive SE event detected by rMATS in the RHOA gene. (a) The 2nd last exon (red box) of RHOA gene model (Basic Gene Annotation from GENCODE 33 lift over to GRCh37), which resides in the negative strand is displayed. Each row depicts a single isoform of the gene. (b) The normalized read coverage of the gene in three replicates of RSEM-simulated RNA-seq for Condition 1. (c) The normalized read coverage of the gene in three replicates of RSEM-simulated RNA-seq for Condition 2. The expression levels (i.e., TPM values inputted to RSEM simulation) of the isoforms of the RHOA gene in (b) and (c) are set to be identical between the simulated conditions in (b) and (c), i.e., the second last exon is not differentially spliced. However, rMATS detected the SE event as differential (FDR < 0.05), while DrSeq did not. The detailed results of DrSeq and rMATS on the SE event are summarized in Tables S1 and S2. (d) A zoomed-in view of (a-c) for the skipped exon event (red box) with re-scaled y-axis.

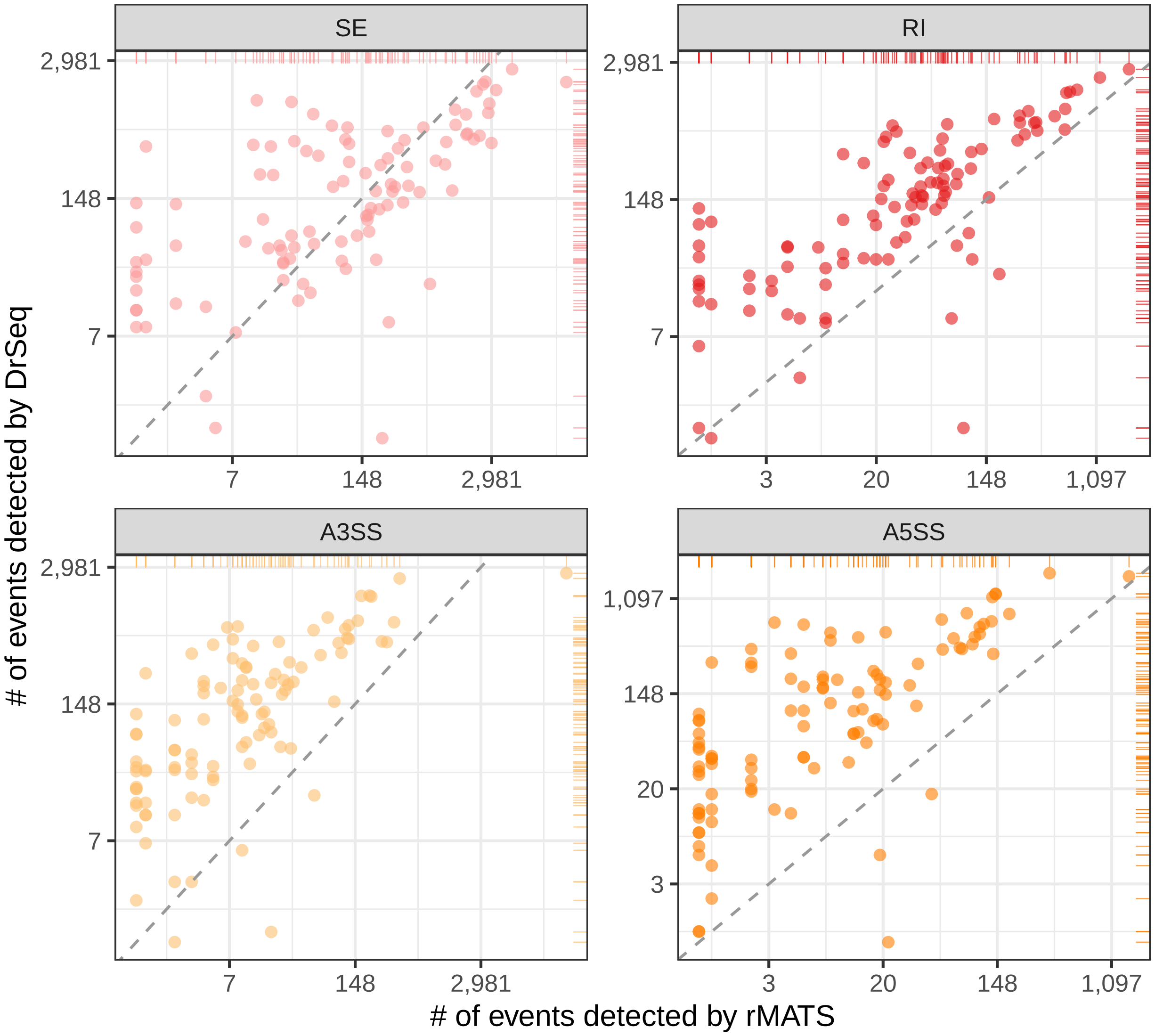

Fig. S13: Comparison of differential AS detection by DrSeq and rMATS in 104 short-hairpin RNA knock-down followed by RNA-seq (with paired control) experiments in K562 cells. Each panel corresponds to one of the four common AS event types (SE, RI, A3SS, and A5SS) reported by both methods. Each point indicates the number of detected differential AS events (FDR < 0.05) in one experiment (i.e., one RBP target) by rMATS (x-axis) and DrSeq (y-axis). The rMATS results were obtained from ENCODE portal (as of February 2020 and without surrogate variable analysis), and were based on rMATS software version 3.2.1.beta and human genome assembly version GRCh37.

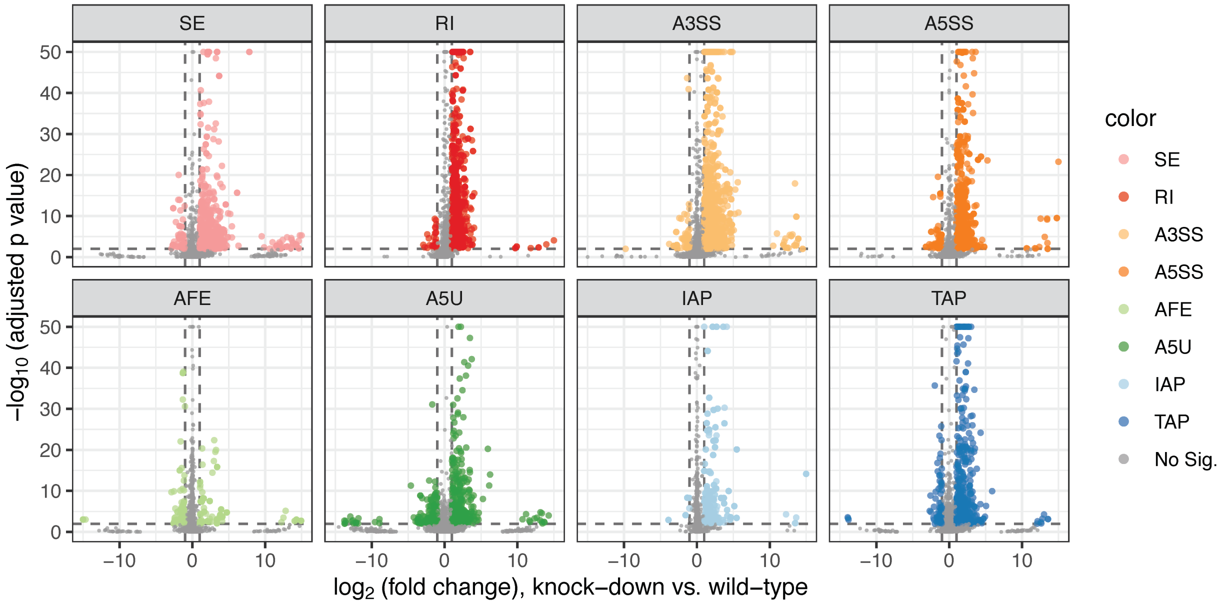

Fig. S14: Volcano plot ($-\log_{10}$ transformed adjusted p-value versus $\log_2$ of fold change) of DrSeq results for AQR, stratified by ATR event types. Horizontal dashed line depicts FDR cut-off level of 0.01 and the vertical lines depict $\log_2$ fold change of 1 in absolute value.

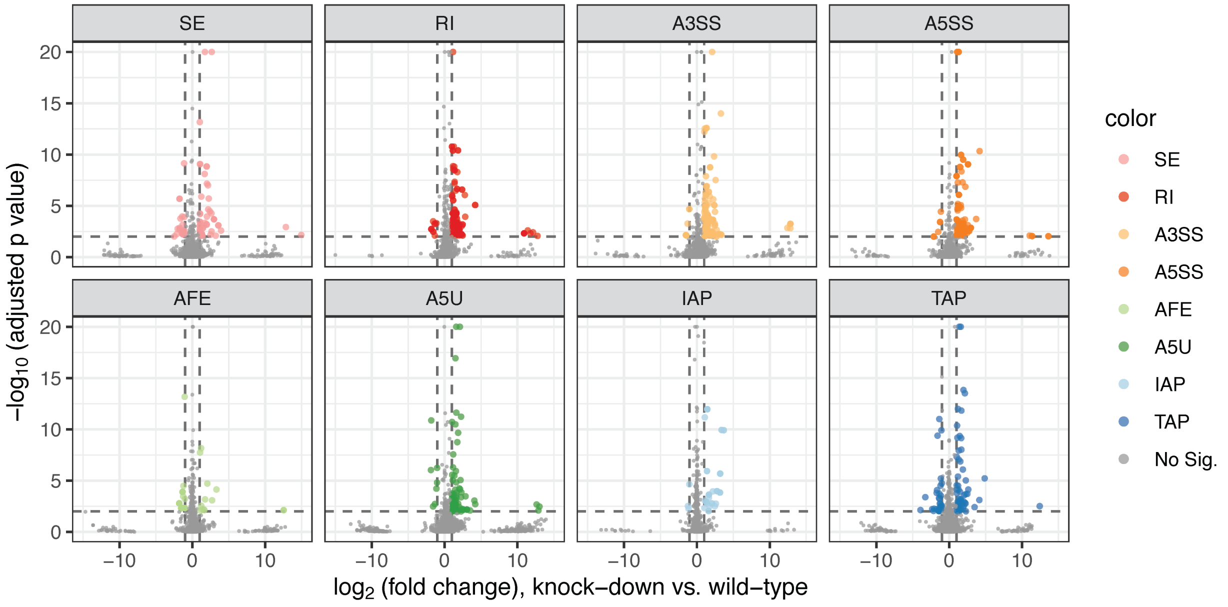

Fig. S15: Volcano plot ($-\log_{10}$ transformed adjusted p-value versus $\log_2$ of fold change) of DrSeq results for SF3B4, stratified by ATR event types. Horizontal dashed line depicts FDR cut-off level of 0.01 and the vertical lines depict $\log_2$ fold change of 1 in absolute value.

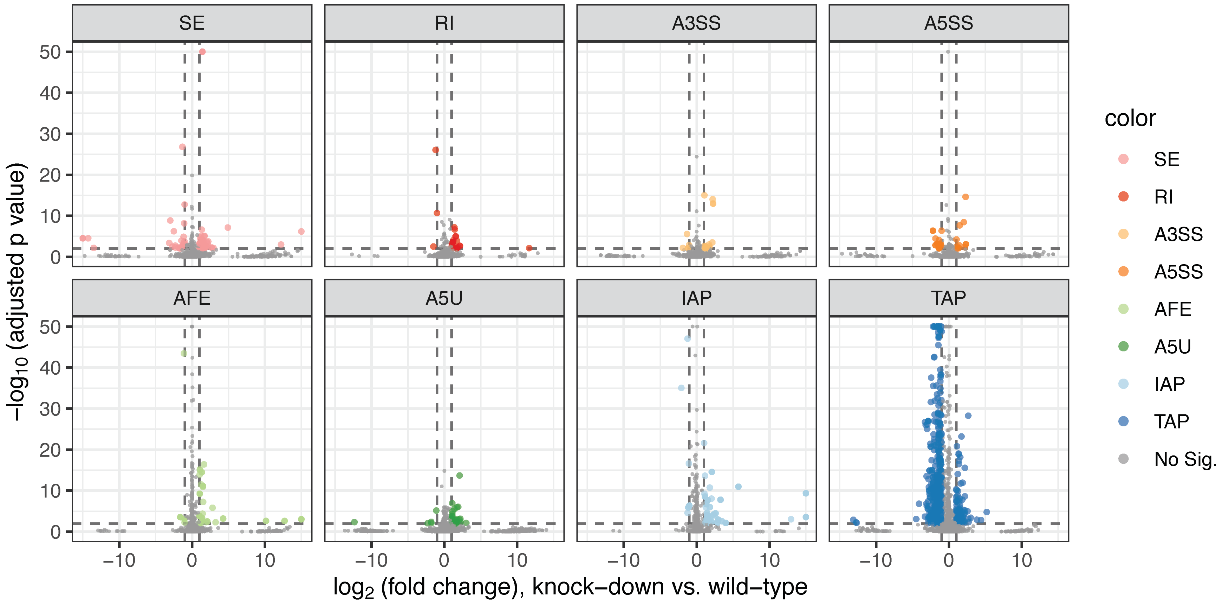

Fig. S16: Volcano plot ($-\log_{10}$ transformed adjusted p-value versus $\log_2$ of fold change) of DrSeq results for CPSF6, stratified by ATR event types. Horizontal dashed line depicts FDR cut-off level of 0.01 and the vertical lines depict $\log_2$ fold change of 1 in absolute value.

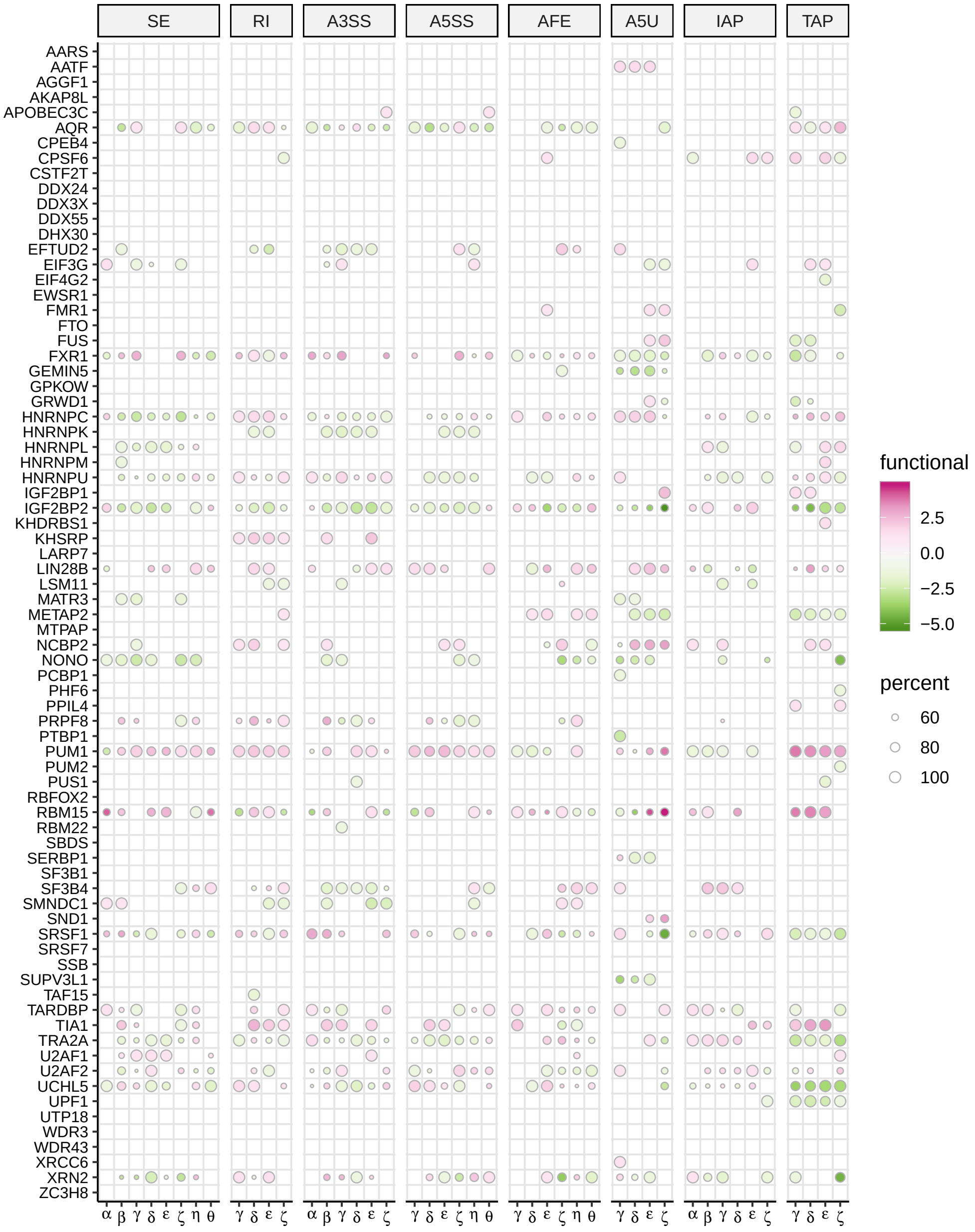

Fig. S17: SURF-identified associations of location features with differential ATR across 73 RBPs (including the ones for which none of the feature locations significantly associated with the DrSeq-inferred differential ATR status). Each row depicts a single RBP and each column represents one location feature, grouped by the corresponding ATR event types (column strips). Each circle symbol in individual cells indicates a significant association at FDR of 0.05. The color of the circles represents inclusion (pink) or exclusion (green) and the fill-in densities are determined by $-\log_{10}$ transformed adjusted p-values from the association testing. For features with dual functions (i.e., binding of RBPs at these location features associates with both inclusion and exclusion), the circle size indicates the percentage of $-\log_{10}$ transformed adjusted p-value for the stronger association relative to the sum of both, i.e., smaller circles indicate similar associations for both inclusion and exclusion.

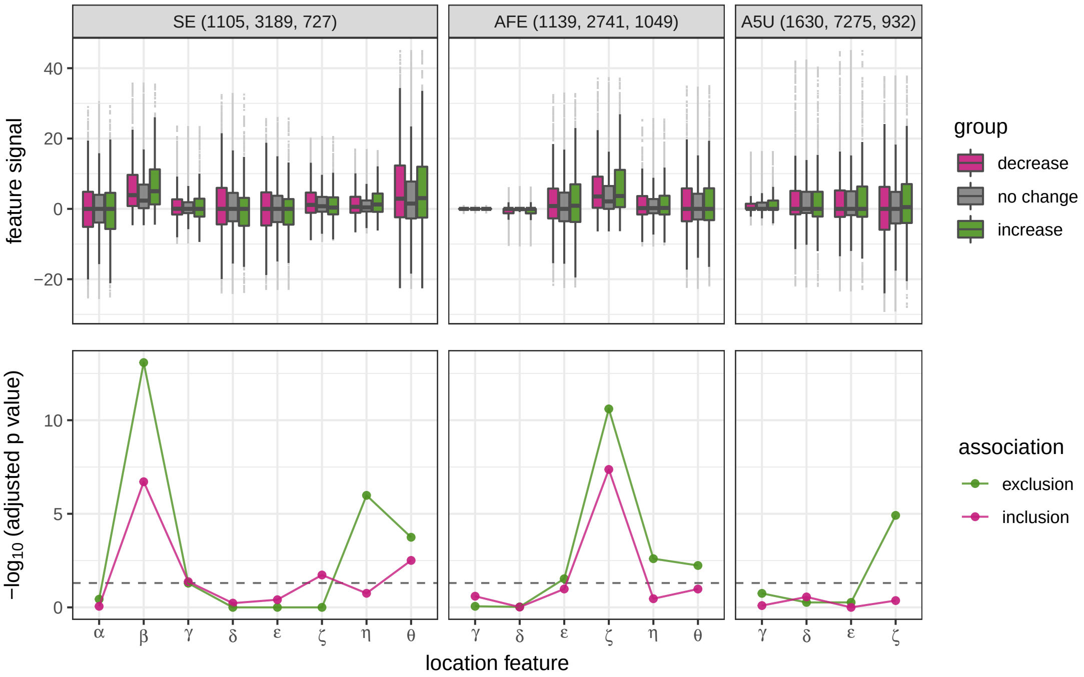

Fig. S18: Functional association (FA) plot of AQR for event types SE, AFE, and A5U. Upper panel box plots display the distributions of feature signals among the three differential ATR groups (decrease, no change, increase). The numbers of ATR events in each group are reported in the parentheses at the top plot strip. The lower panels depict the $-\log_{10}$ transformed p-values for each tested association after multiplicity correction. The dashed lines indicate the FDR level of 0.05.

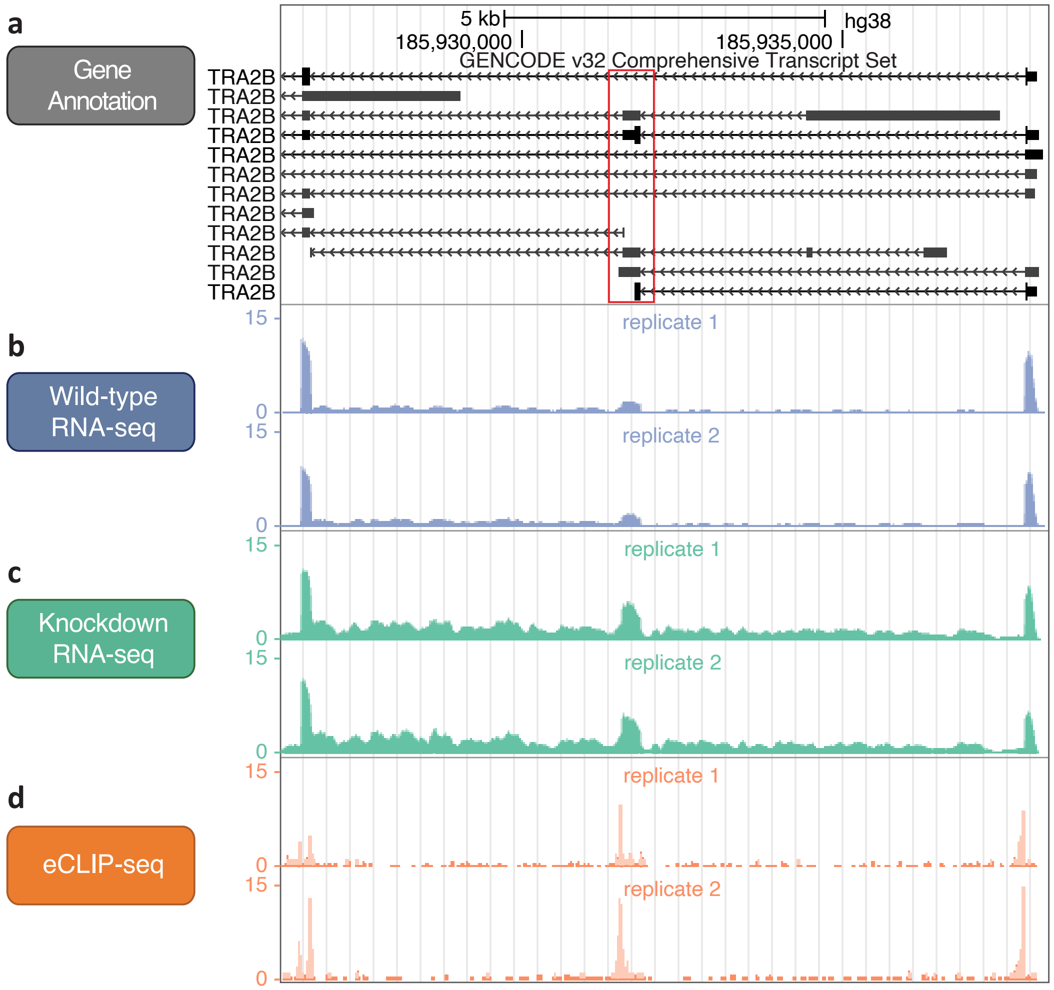

Fig. S19: An included exon in the TRA2B gene upon AQR knock-down. (a) The 2nd exon (red box) of TRA2B gene model (GENCODE version 32) which resides in the minus strand. Each row depicts a single isoform of the gene. (b) The normalized read coverage in two replicates of wild-type RNA-seq. The normalized read coverage in two replicates of shRNA AQR knock-down followed by RNA-seq. The 2nd exon exhibits an increased relative exon usage when comparing the knock-down and wide-type conditions. (d) The normalized read coverage in two replicates of AQR eCLIP-seq.

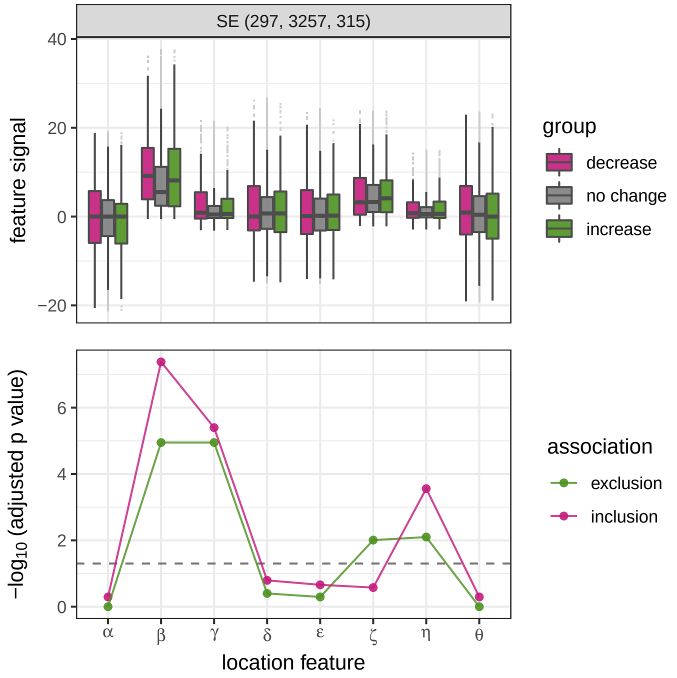

Fig. S20: FA plot of PRPF8 for event type SE. See Fig. S18 for more information.

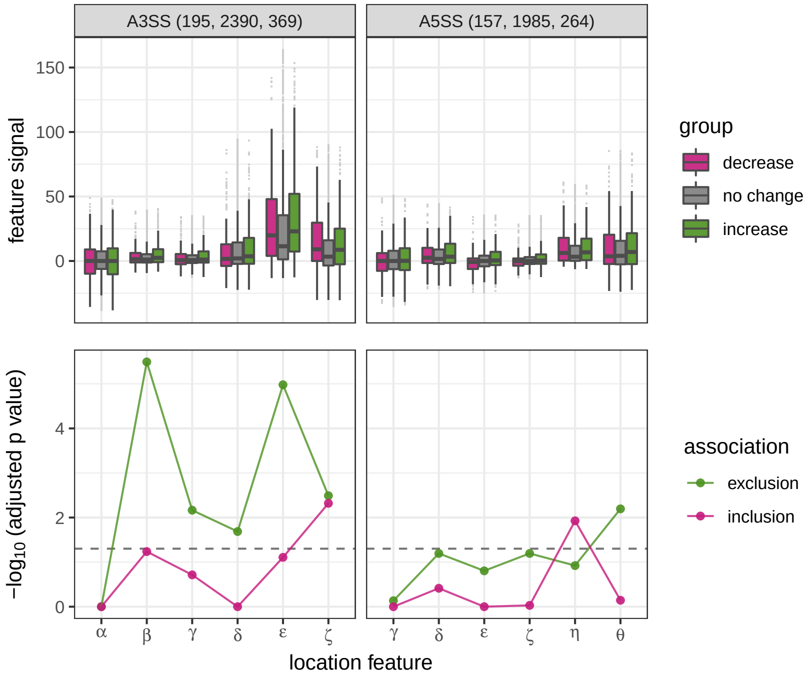

Fig. S21: FA plot of SF3B4 for event types A3SS and A5SS. See Fig. S18 for more information.

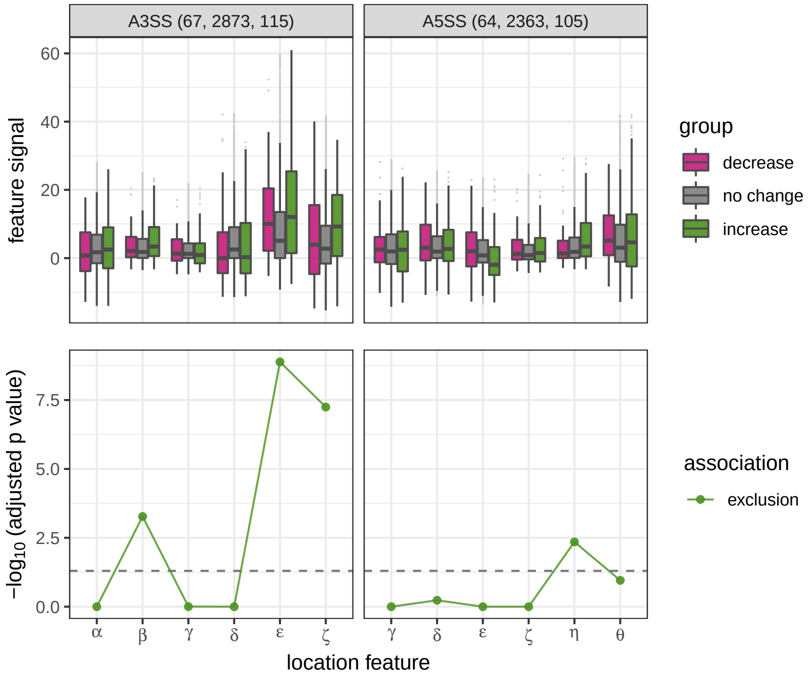

Fig. S22: FA plot of SMNDC1 for event types A3SS and A5SS. See Fig. S18 for more information.

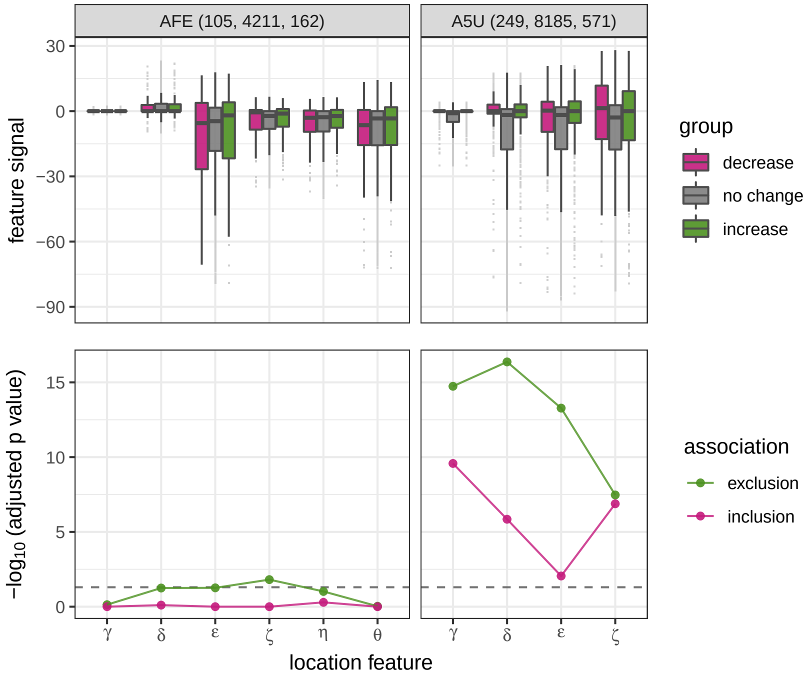

Fig. S23: FA plot of GEMIN5 for event types AFE and A5U. See Fig. S18 for more information.

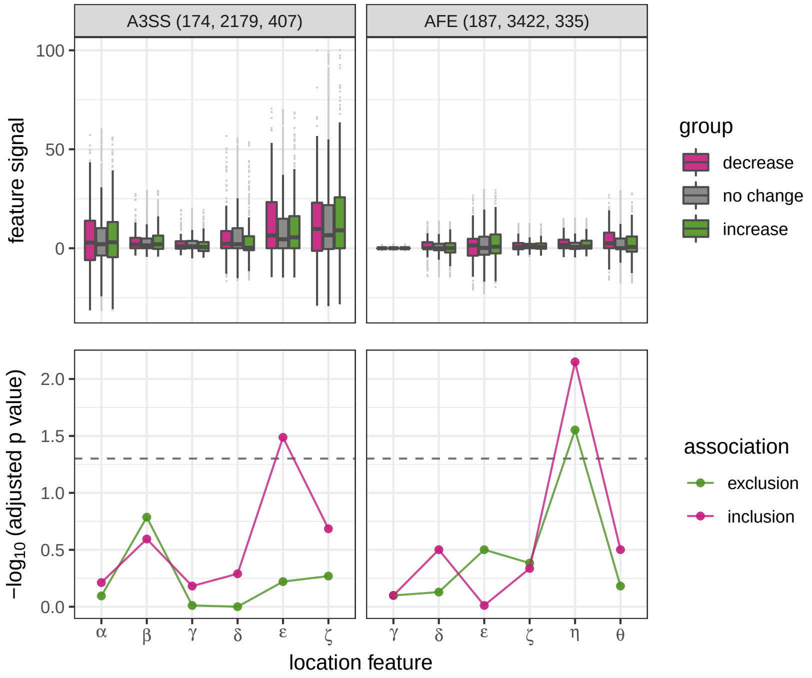

Fig. S24: FA plot of U2AF1 for event types A3SS and AFE. See Fig. S18 for more information.

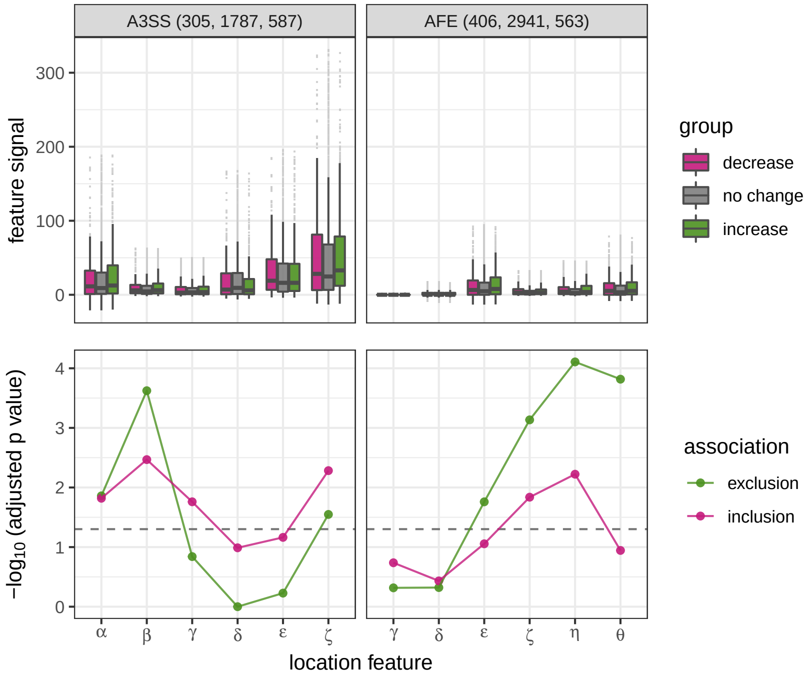

Fig. S25: FA plot of U2AF2 for event types A3SS and AFE. See Fig. S18 for more information.

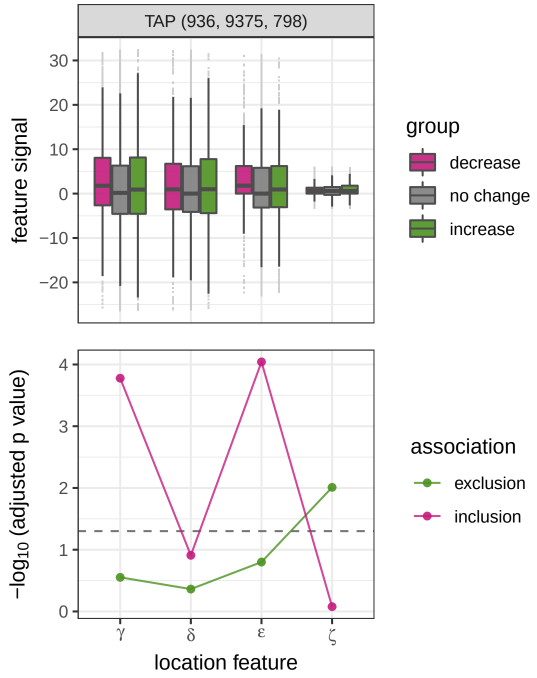

Fig. S26: FA plot of CPSF6 for event type TAP. See Fig. S18 for more information.

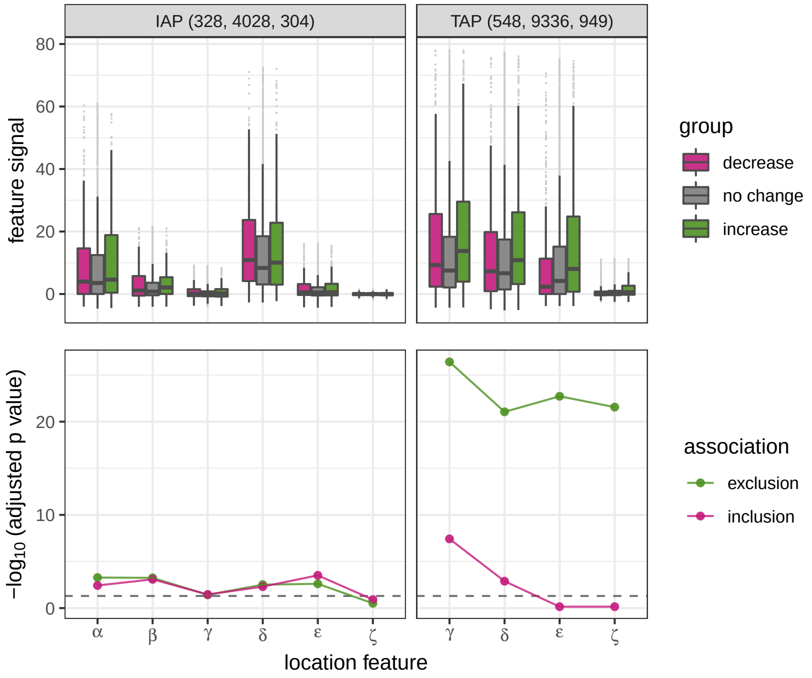

Fig. S27: FA plot of UCHL5 for event types IAP and TAP. See Fig. S18 for more information.

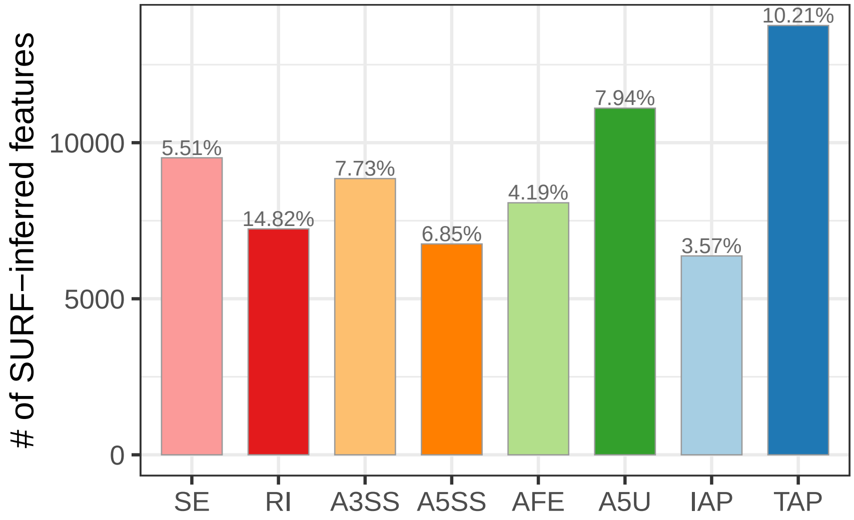

Fig. S28: Numbers of SURF-inferred features (i.e., genomic locations for which an RBP binding is associated with differential ATR) for each ATR event category. The percentage (%) of SURF-inferred location features among all possible location features of the ATR event type is listed above each bar.

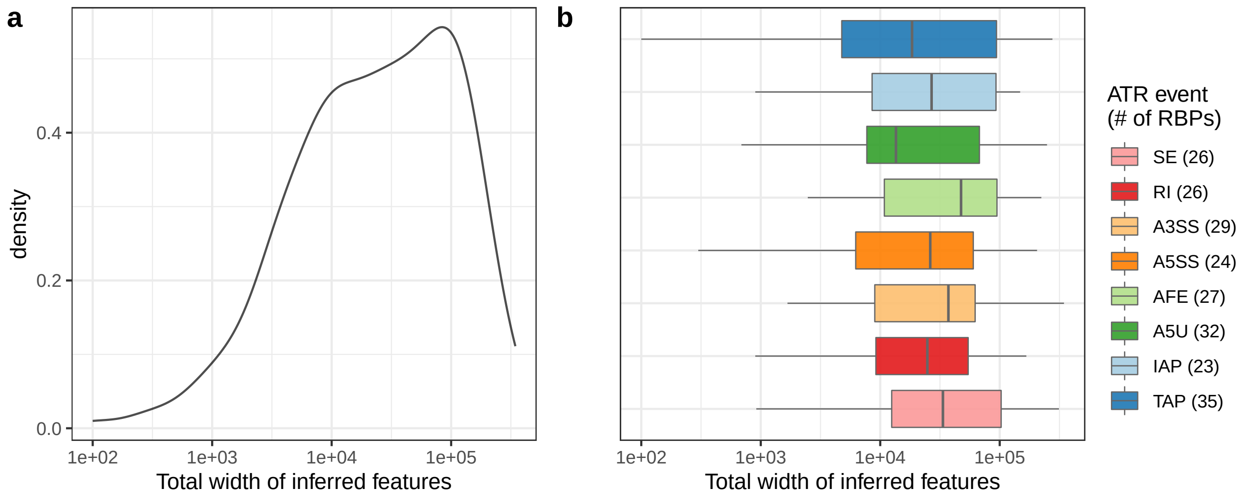

Fig. S29: Distribution of the total length of SURF-inferred location features. Left panel density plot depicts the distribution over 53 individual RBPs. Right panel box plots depict the distributions stratified by ATR event types. For each event type, the number of RBPs with SURF-inferred location features is reported in parentheses with color legend.

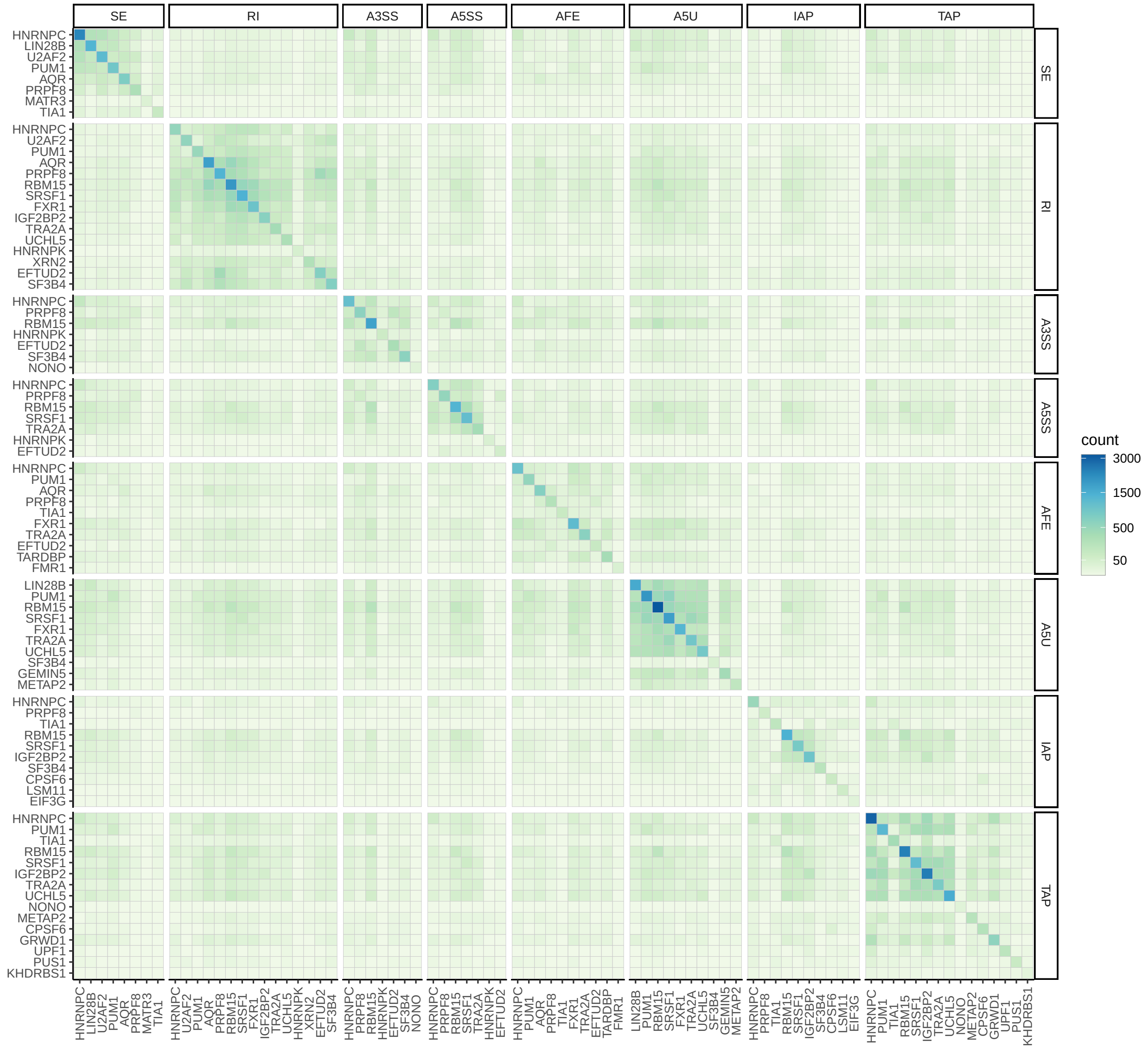

Fig. S30: Clustering of RBP-ATR event types based on their numbers of shared SURF-inferred location features. The heatmap depicts a total of 82 RBP-ATR event types. Each entry reports the overlap between the two RBP-event type combinations. The diagonal color shade indicates the total number of SURF-inferred location features associated with each RBP-ATR event type combination. The off-diagonal color shades depict the numbers of shared location features between pairs of RBP-ATR event type combinations.

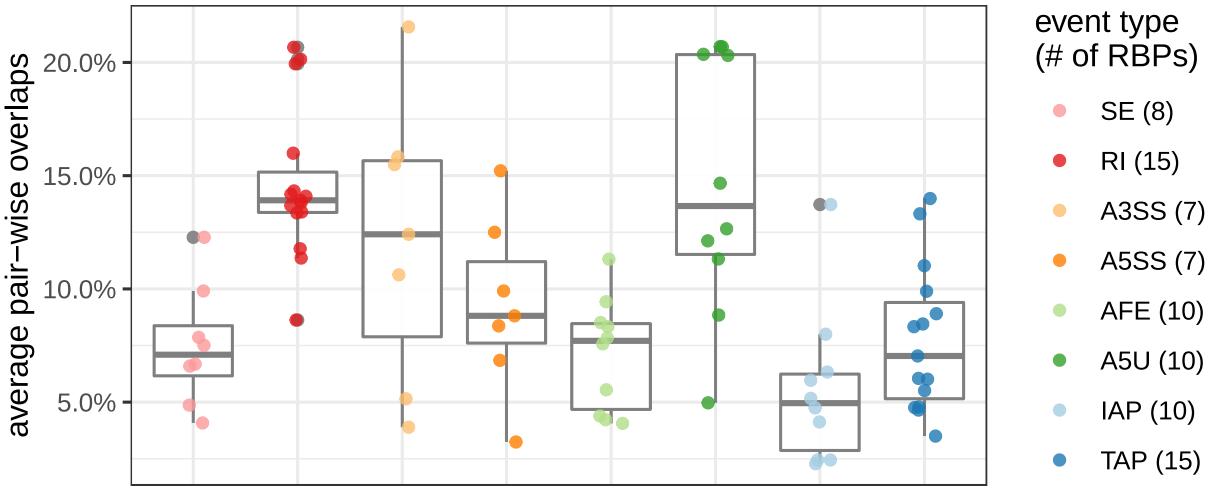

Fig. S31: Summary of the extent of overlap between the SURF-inferred location feature sets of 82 RBP-ATR event type combinations. For each RBP-ATR event type combination, the corresponding SURF-inferred location feature set is compared with location feature sets of other RBPs within the same ATR event type. The average overlap is quantified by the average of percentages of its location features that are shared by other RBPs within the same event type. For each event type, the number of location feature sets (i.e., the number of RBPs) is reported in parentheses in the color legend.

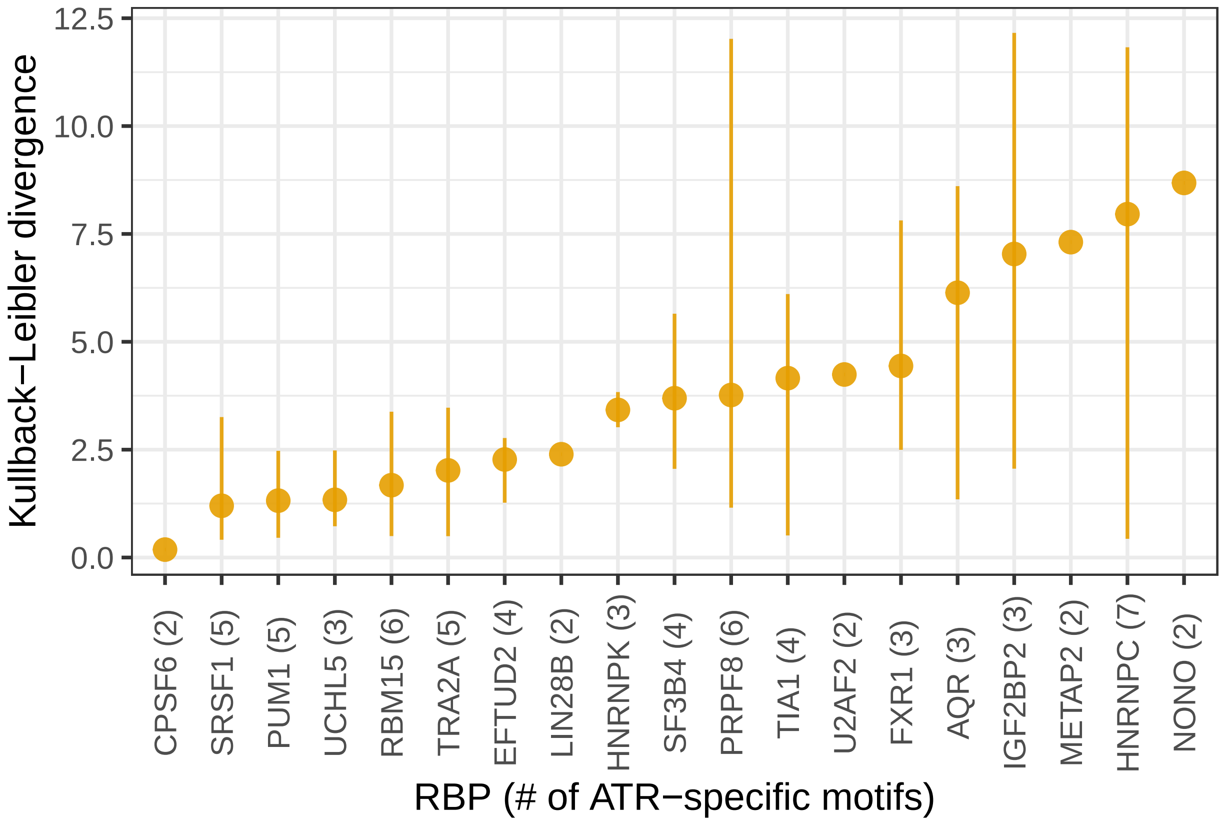

Fig. S32: Diversity of multiple motifs identified for individual RBPs. Position weights matrices of the motifs (E-value < 0.01) identified by *de novo* sequence analysis of SURF-identified location features of RBPs across event types are compared with Kullback-Leibler (KL) divergence (y-axis). For each RBP, the number of significant motifs is reported in parenthesis in the x-axis label For each RBP, average diversity of all pairs of learnt motifs is reported along with the range (depicted by the vertical lines) indicating the minimum and maximum deviations observed across all pairs of motifs for the RBP.

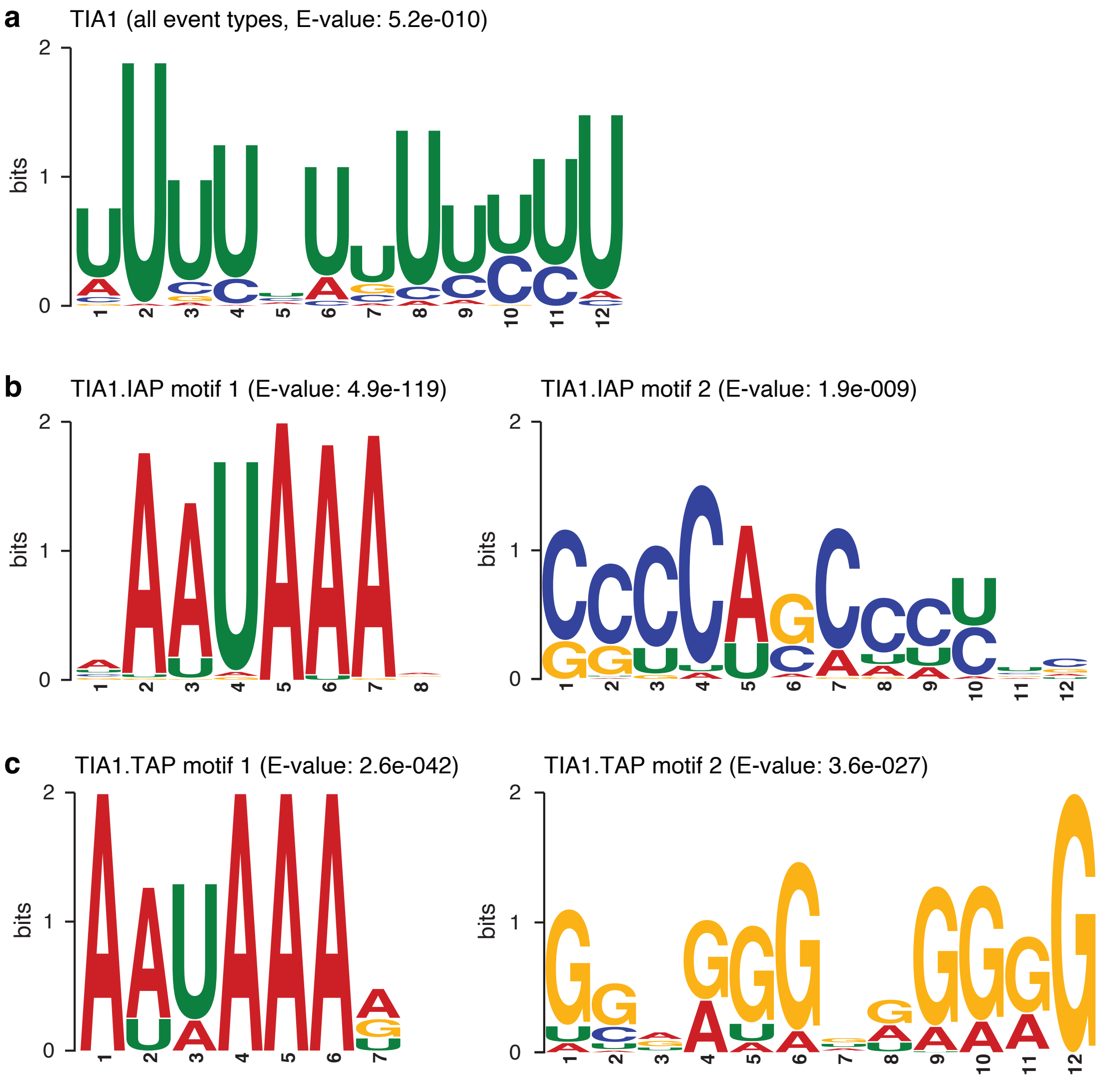

Fig. S33: (a) Sequence logo of motif identified in 348 SURF-inferred location features of TIA for ATR. (b) Sequence logos of the two motifs identified in 161 SURF-inferred location features for TIA1 in regulation of IAP events. (c) Sequence logos of the two motifs identified in 144 SURF-inferred location features for TIA1 in regulation of TAP events. The total height of the letters depicts the information content of the position in bits.

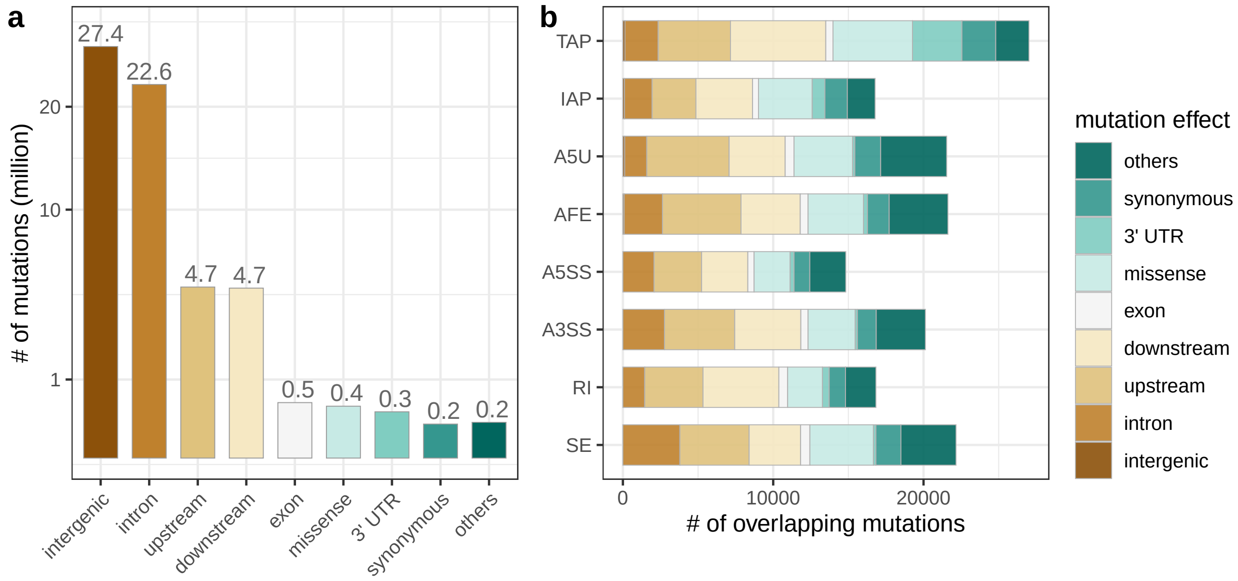

Fig. S34: (a) Genomic location annotations of somatic mutations from ICGC (non-US) projects. The percentage of mutations in each category is listed above each bar. (b) The composition of ICGC mutation effects overlapping with SURF-inferred location features based on 54 RBPs. The class of intergenic mutations has fewest overlaps.

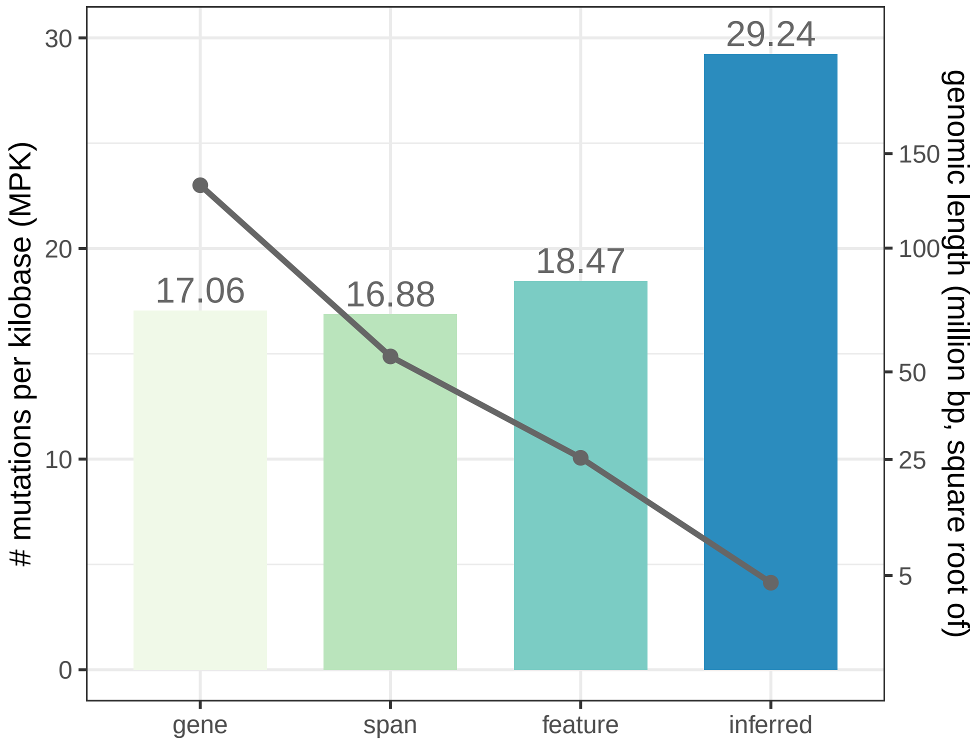

Fig. S35: Mutations per kilobase (MPK) of SURF-inferred features (inferred) and three control genomic sets (gene, span, and feature). The bars depict the MPK quantified with somatic mutations from the TCGA project. The solid line with the right-hand side axis depicts the total length of the genomic regions considered.

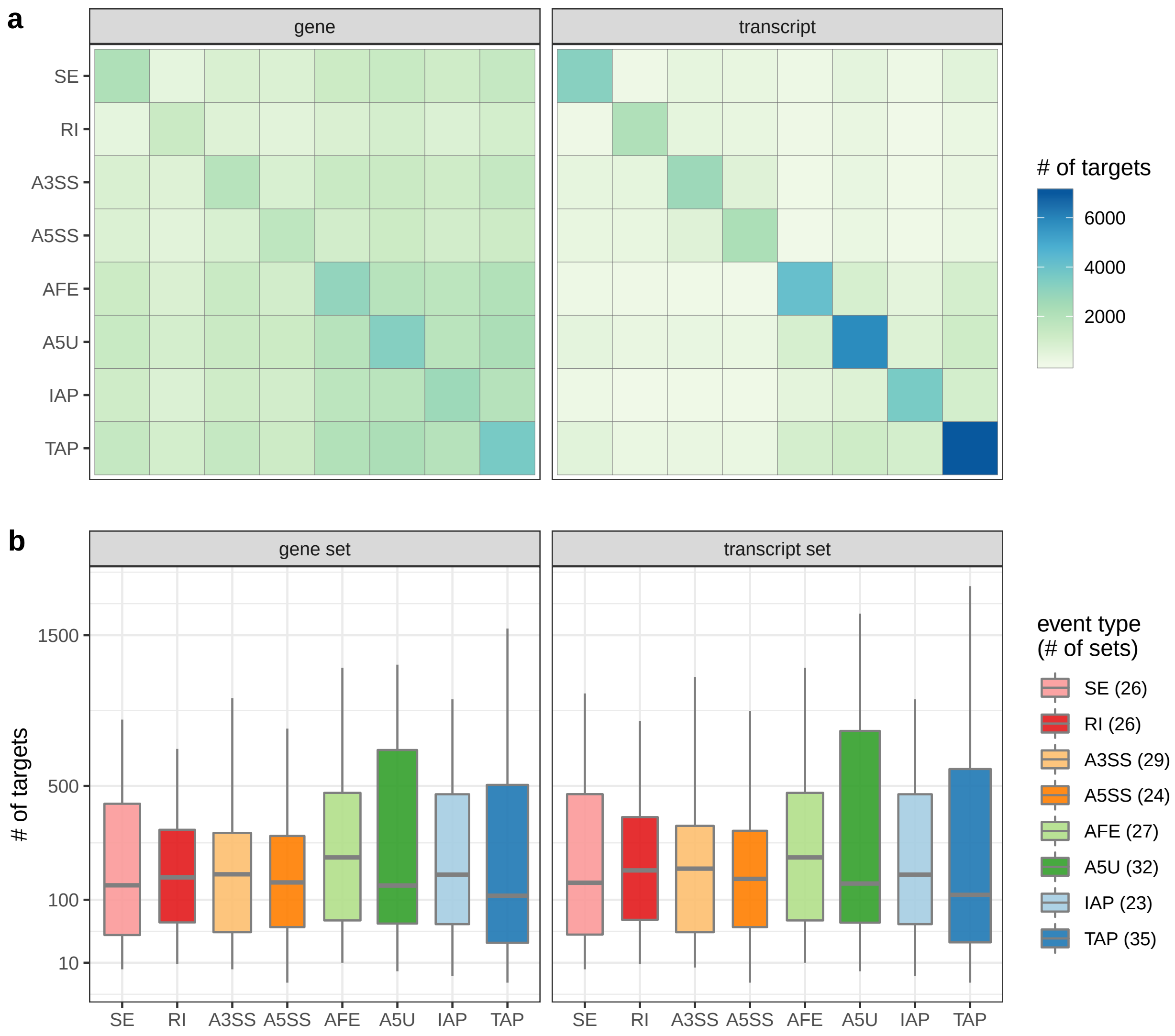

Fig. S36: The number of SURF-inferred target genes and target transcripts harboring SURF-inferred location features of 52 RBPs. (a) The number of target genes (left) and transcripts (right) shared between pairs of ATR event types. The number of targets belonging to each ATR event types is depicted in diagonal. (b) The size of target gene sets (left) and transcript sets (right) grouped by associated ATR event type. For each event type, the number of target sets is reported in parentheses in the color legend.

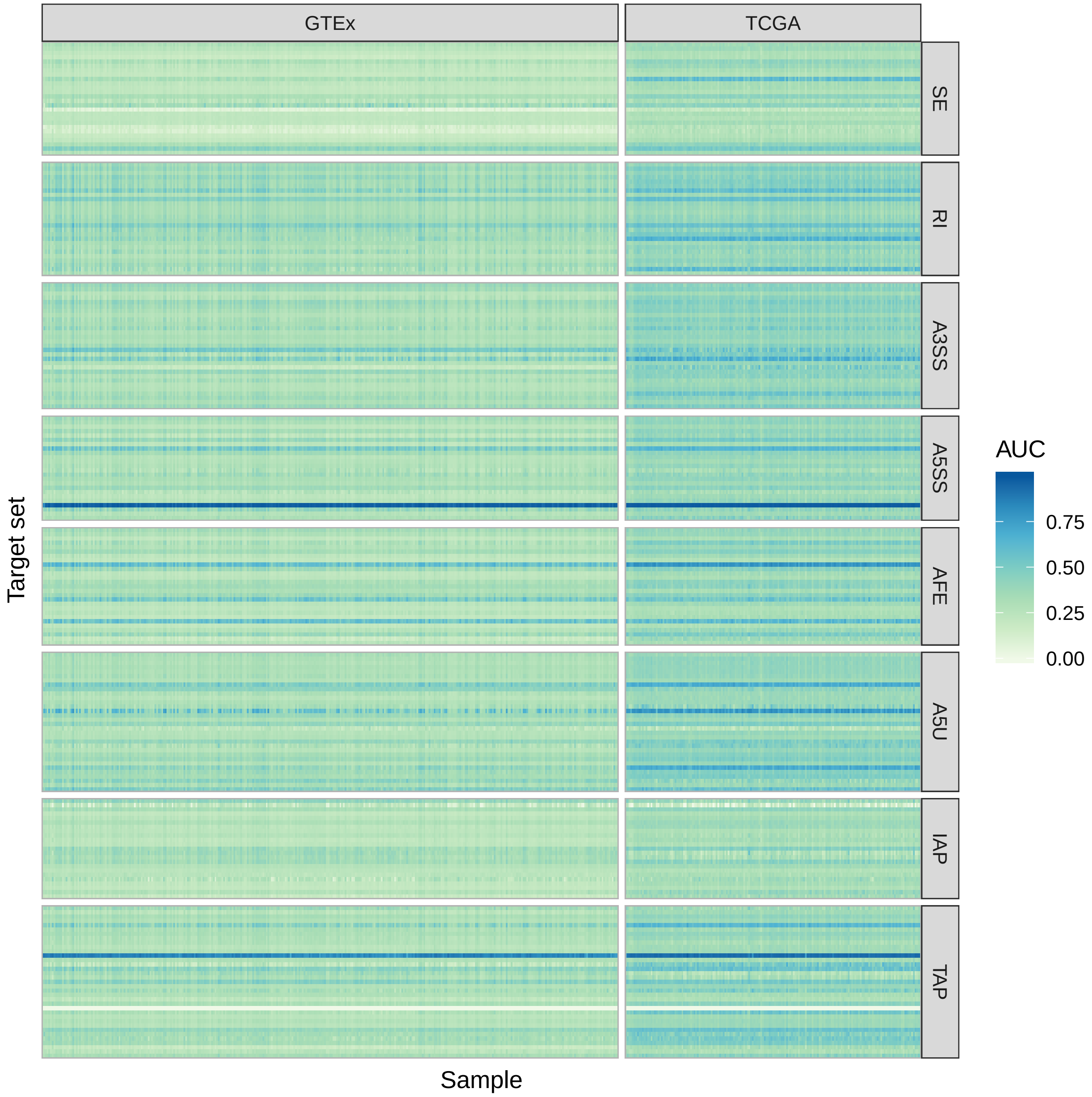

Fig. S37: Heatmap of the observed AUC for 222 gene sets (row) in 337 GTEx whole blood samples and 173 TCGA LAML samples (column). Row strips indicate the eight corresponding ATR event types of the gene sets.

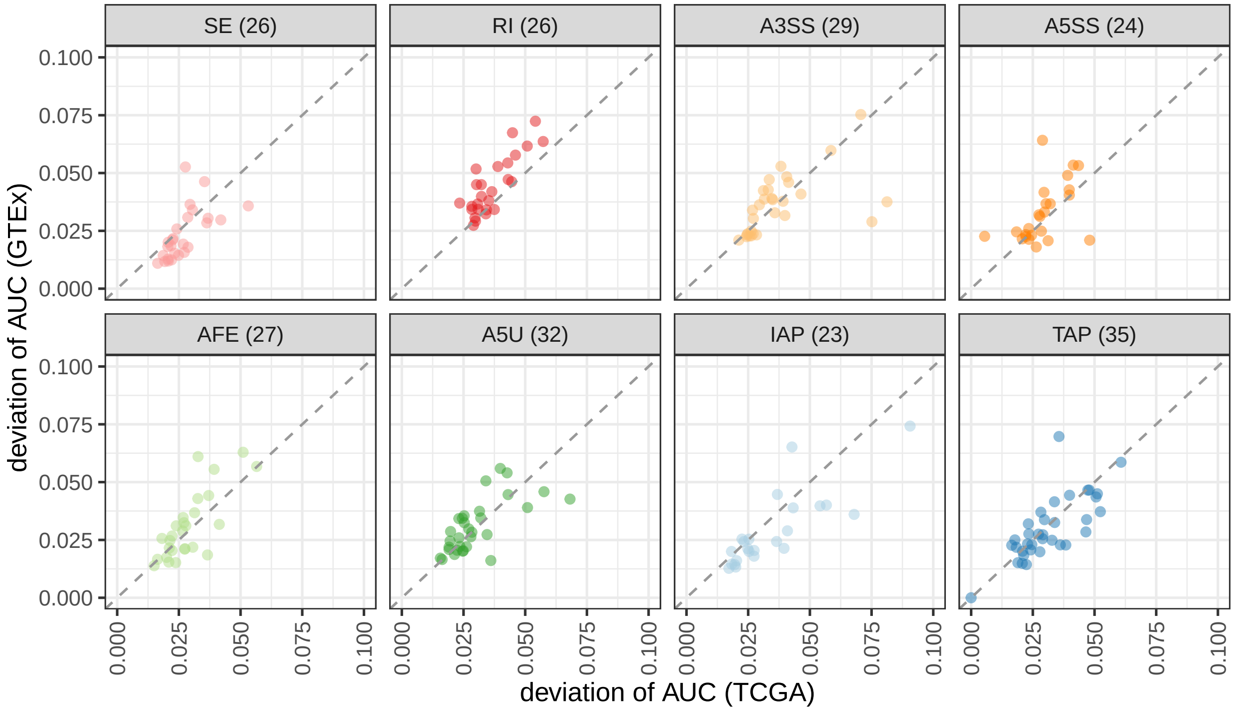

Fig. S38: The variation of empirical AUC for 222 target gene sets harboring SURF-identified location features, stratified by associated ATR event type. Each point indicates, for one gene set, the standard deviation of AUC in 173 LAML (TCGA) samples and in 337 whole blood (GTEx) samples. The diagonal dashed line in each panel indicates equal variance between two projects. For each event type, the number of target gene sets is reported in parentheses within the top panel strips.

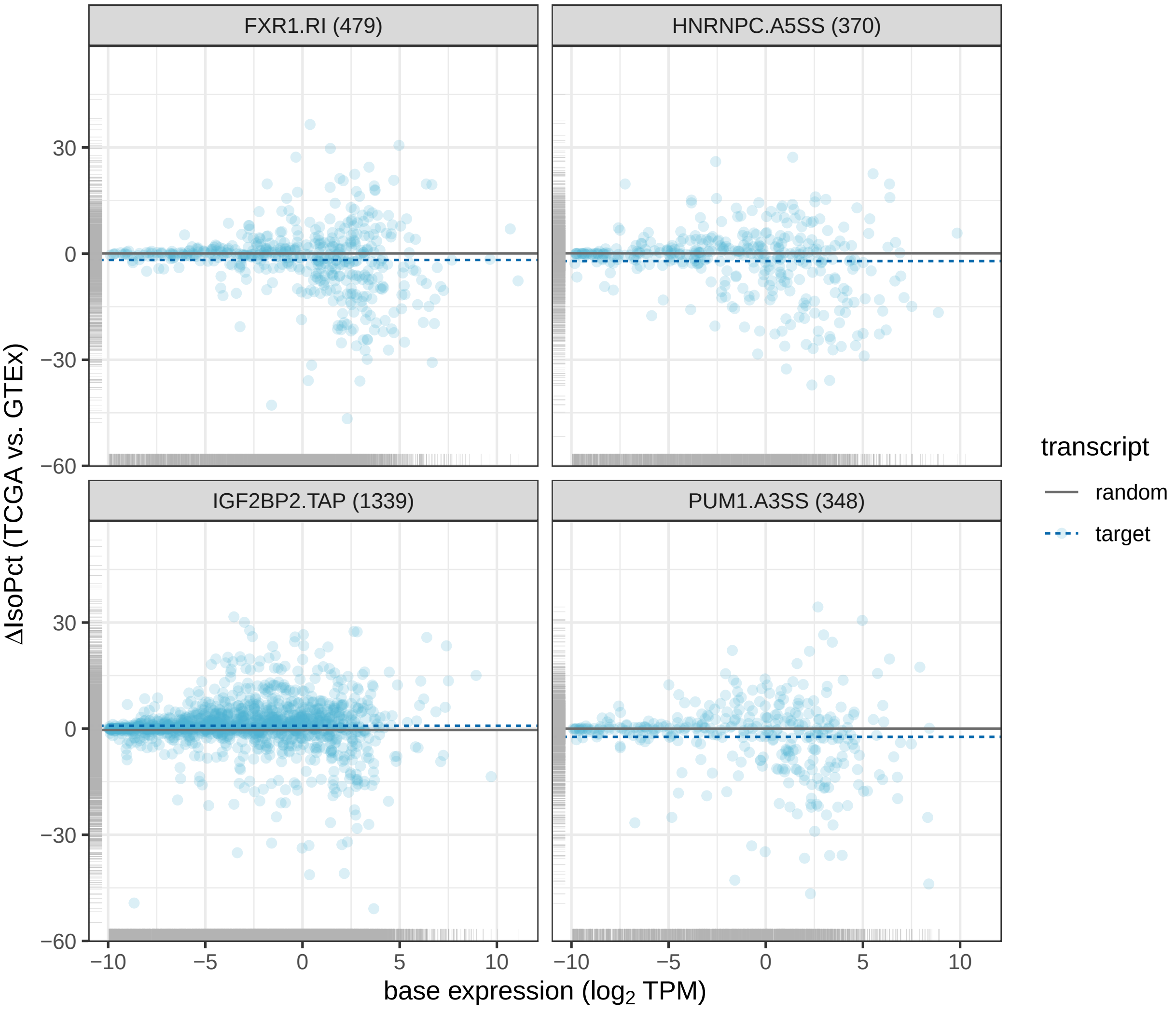

Fig. S39: Comparison of target transcript expression changes in GTEx whole blood and TCGA LAML RNA-seq samples. The four panels depict SURF-inferred transcript targets of (i) FXR1 in RI, (ii) HNRNPC in A5SS, (iii) IGF2BP2 in TAP, and (iv) PUM1 in A3SS. Each point displays the base expression (in $\log 2$ transformed TPM) and the difference in isoform percent (ΔIsoPct) from normal (GTEx) to tumor (TCGA) samples. The number of transcripts in each target set is reported in parentheses in the top panel strips. A dashed blue line indicates the average ΔIsoPct across the target transcript set, and a solid grey line indicates the average ΔIsoPct across the randomized control transcript sets.

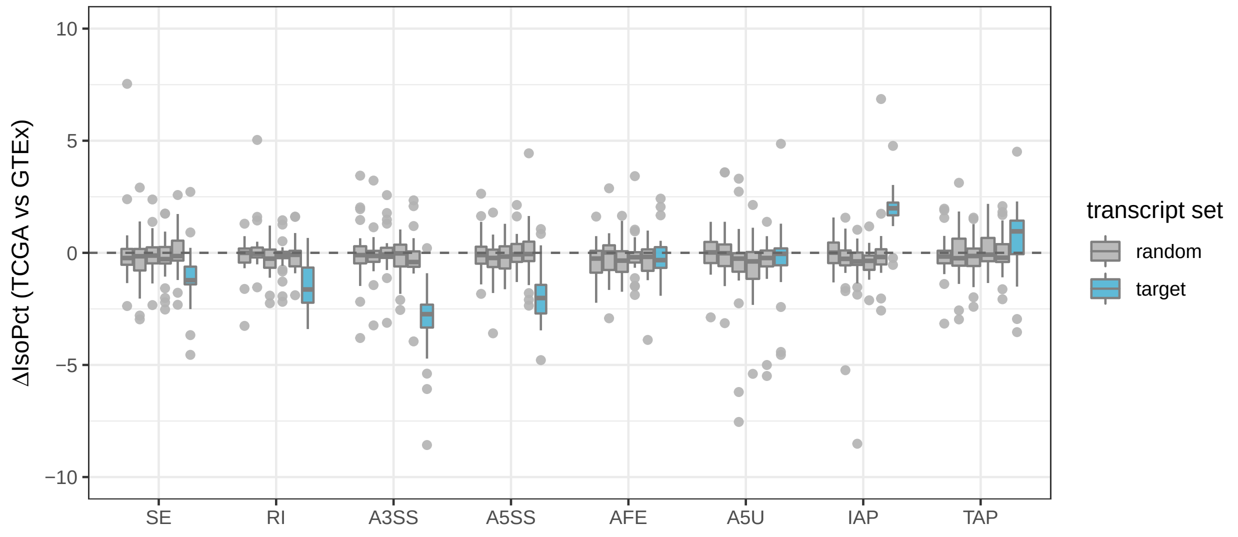

Fig. S40: Comparison of the average changes in isoform percentages of transcript sets between TCGA LAML and GTEx whole blood samples, with multiple replicates of control transcript sets, across ATR event types. Boxplots are over either target transcript sets (blue) or randomized control transcript sets (grey, 5 sets of controls for each event type). The differences in isoform percentages (ΔIsoPct) between TCGA LAML and GTEx whole blood samples are averaged within individual transcript sets. 222 transcript sets are depicted in one of eight associated ATR event types.

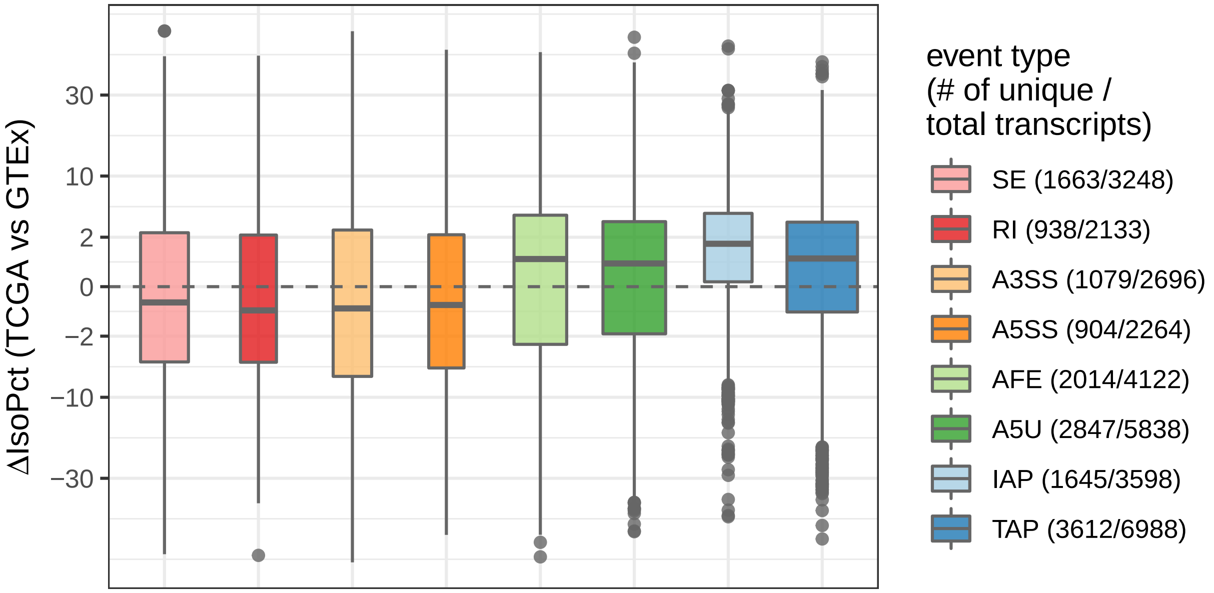

Fig. S41: Comparison of ΔIsoPct (see Fig. S40) for a subset of SURF-inferred target transcripts that uniquely associated with only one ATR event type. The numbers of transcripts uniquely associating with each event type are reported in parentheses in the color legend along with the total numbers of transcripts of the event types and are also depicted by the width of the box plots. The differences in the ΔIsoPct of the four classes of AS events (SE, RI, A3SS, and A5SS) are not statistically significant (adjusted p-values > 0.05 in pairwise t-tests). Similarly, the differences between the two ATI event types (AFE and A5U) are not statistically significant (adjusted p-value > 0.05). Remaining pairwise comparisons of ΔIsoPct values between ATR event types (e.g., between AS and APA event types) are significantly different (adjusted p-values < 0.01)

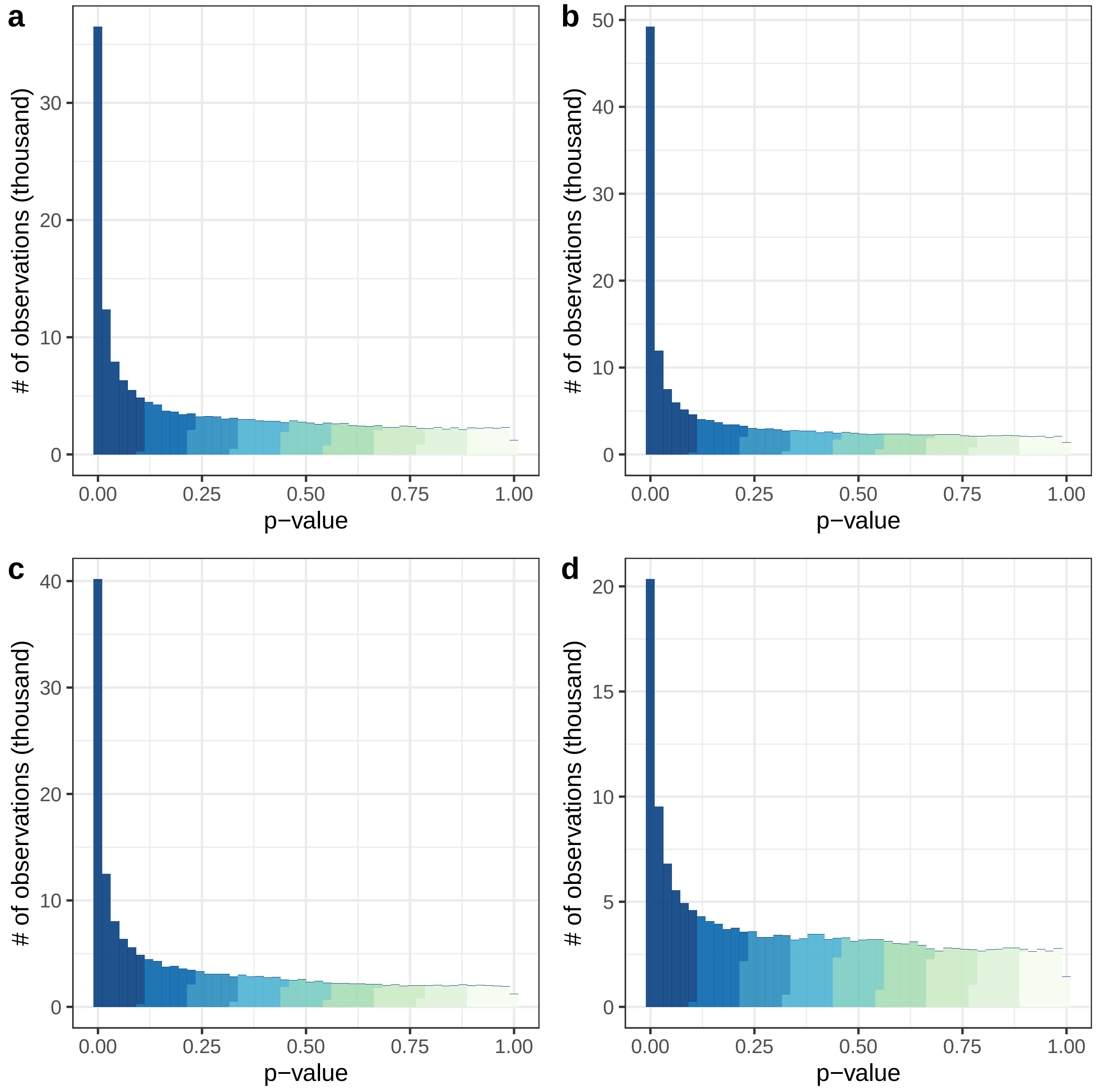

Fig. S42: Histogram of raw p-values from the differential ATR analysis (DrSeq) of ENCODE datasets (a) AQR, (b) SRSF1, (c) CPSF6, and (d) SF3B4. Histograms display a mixture of uniform distribution on $[0, 1]$ and a point mass at zero, suggesting well-calibrated p-values.

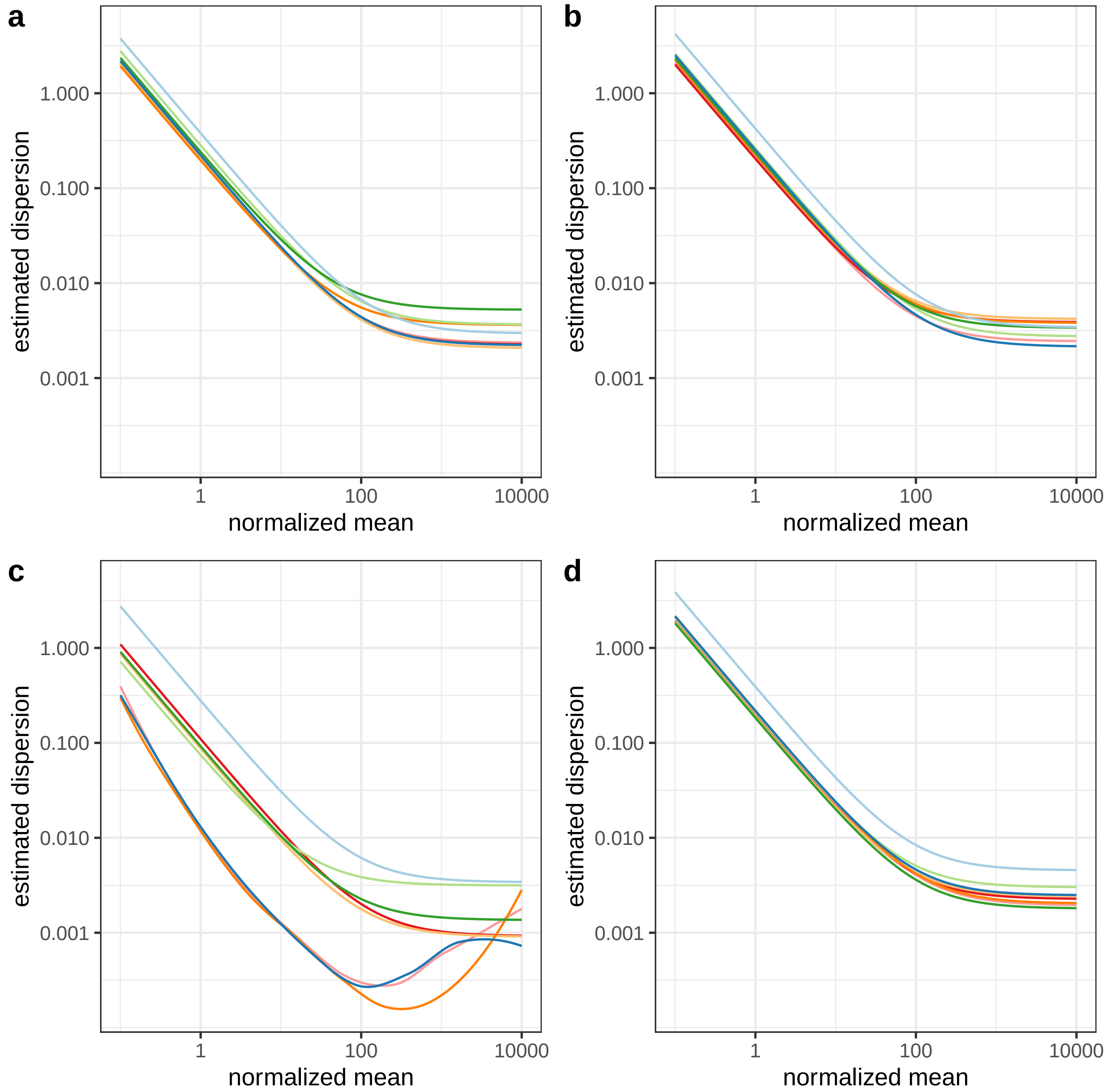

Fig. S43: Estimated mean-dispersion functions from DrSeq analysis of RNA-seq datasets (ENCODE) with RBP targets (a) AQR, (b) SRSF1, (c) CPSF6, and (d) SF3B4. Each line corresponds to an ATR event type from Fig. S1.

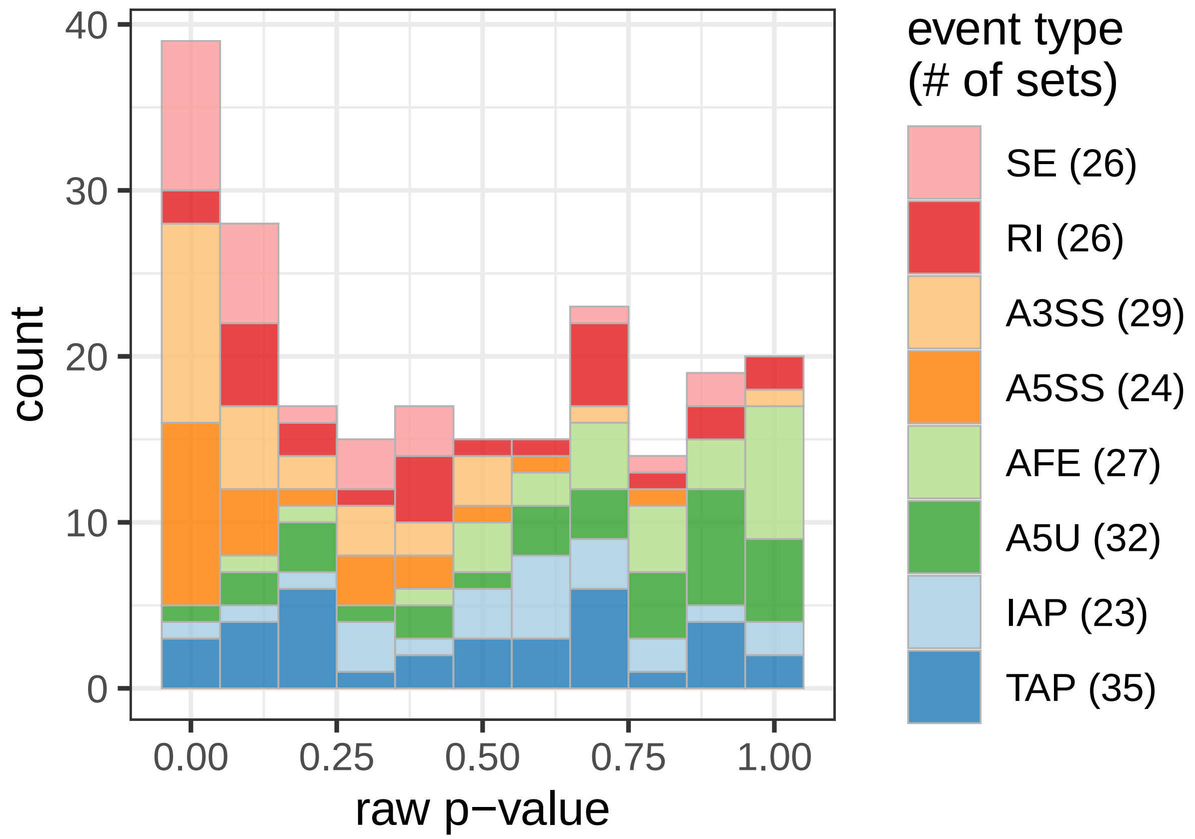

Fig. S44: Histogram of raw p-values from the application of SURF discovery module to 222 target transcript sets. The transcript sets are colored with the associated ATR event types. For each event type, the number of transcript sets is reported in parentheses in the color legend.

Supplementary Tables

Table S1: Summary of DrSeq results for the differential skipping of the 2nd last exon in the RHOA gene. Counts across replicates of each condition are separated by semicolons. Fig. S12 displays a genome browser view of the normalized RNA-seq read coverage.

	DrSeq results
gene identifier	ENSG00000067560
gene name	RHOA
event defining transcript identifier	ENST00000418115
chromosome name	chr3
strand	-
skipping exon (start-end)	49,398,361-49,398,499
event body read counts (condition 1)	142; 111; 98
event body read counts (condition 2)	265; 27; 65
gene read counts (condition 1)	17,260; 17,203; 16,399
gene read counts (condition 2)	30,915; 17,744; 23,840
base normalized read counts	115.222
estimated dispersion parameter	0.150
estimated REU coefficient (condition 1)	17.336
estimated REU coefficient (condition 2)	14.765
$\log_2$ REU fold change (condition 2 vs 1)	-0.487
significance of differential REU (p-value)	0.129
adjusted p-value	0.773

Table S2: Summary of rMATS results for the differential skipping of the 2nd last exon in the RHOA gene. Counts across replicates of each condition are separated by semicolons. Fig. S12 displays a genome browser view of the normalized RNA-seq read coverage.

	rMATS results
gene identifier	ENSG00000067560
gene name	RHOA
chromosome name	chr3
strand	-
skipping exon (start-end)	49,398,360-49,398,499
inclusion junction counts (condition 1)	210; 146; 139
skipping junction counts (condition 1)	1594; 1501; 1425
inclusion junction counts (condition 2)	369; 39; 93
skipping junction counts (condition 2)	2711; 1613; 2156
length of inclusion form	239
length of skipping form	100
significance of splicing difference (p-value)	2.81e-06
FDR (adjusted p-value)	1.05e-04
inclusion level (condition 1)	0.052; 0.039; 0.039
inclusion level (condition 2)	0.054; 0.01; 0.018
inclusion level difference (condition 1 vs. 2)	0.016

Table S3: Number of false positives of the SURF analysis module 2 (functional association testing). The analysis module 2 was repeated for each of 100 replicates with permuted differential ATR labels. The average numbers of false positives, with target FDR threshold of 0.01, 0.05, and 0.1 after multiplicity correction across all RBPs and ATR event types, are reported. For each FDR threshold, the standard deviation (std. dev.) of the 100 observed numbers is listed besides.

FDR threshold	average # of false positives	std. dev.
0.01	0.73	0.886
0.05	3.88	2.38
0.10	8.52	3.80

Table S4: The fold change in MPK among four genomic sets – genes, spanning regions of ATR events, location features, and SURF-inferred features – from the ICGC (non-US) projects. For each pair of genomic sets, the observed fold change for the genomic set in column over the genomic set in row is listed in the table. The corresponding p-value of exact Poisson test for each fold change is reported in parentheses.

	span	feature	inferred
gene	1.21 (1.05×10^{-2})	1.24 (4.20×10^{-2})	1.50 (3.00×10^{-4})
span	/	1.02 (3.19×10^{-4})	1.23 (6.12×10^{-3})
feature	/	/	1.20 (3.68×10^{-3})

Table S5: The fold change in MPK among four genomic sets – genes, spanning regions of ATR events, location features, and SURF-inferred features – from the TCGA project. For each pair of genomic sets, the observed fold change for the genomic set in column over the genomic set in row is listed in the table. The corresponding p-value of exact Poisson test for each fold change is reported in parentheses.

	span	feature	inferred
gene	0.99 (0.629)	1.075 (1.22×10^{-5})	1.65 (2.23×10^{-3})
span	/	1.08 (1.05×10^{-12})	1.66 (8.52×10^{-6})
feature	/	/	1.151 (3.88×10^{-9})

Table S6: The activity levels of SURF-identified transcript sets in TCGA LAML and GTEx whole blood samples. SURF-inferred transcription sets corresponding to 222 RBP × event combinations, from which 15 sets with the number of transcripts (size) fewer than 10 are excluded. For each transcript set, the p-value of differential activity between normal (GTEx) and tumor (TCGA) samples, after multiplicity correction using BH procedure, are reported (adj. p-value). The significance codes – $***$: adjusted p-value<0.001, $**$: adjusted p-value <0.01, $*$: adjusted p-value <0.05, $\cdot$: adjusted p-value <0.1 – are also marked.

	factor	event	size	AUC(GTEx)	AUC(TCGA)	adj. p-value	significance
1	AQR	SE	481	0.1500	0.1716	0.4050	
2	EFTUD2	SE	20	0.2579	0.2476	1.0000	
3	EIF3G	SE	92	0.1676	0.2204	0.3863	
4	FXR1	SE	513	0.1085	0.1548	0.0000	***
5	HNRNPC	SE	931	0.0783	0.1103	0.0028	**
6	HNRNPL	SE	25	0.0867	0.1855	0.0727	.
7	HNRNPM	SE	11	0.0000	0.0915	0.2556	
8	HNRNPU	SE	146	0.1513	0.1918	0.8046	
9	IGF2BP2	SE	403	0.0947	0.0957	1.0000	
10	LIN28B	SE	674	0.0747	0.0959	0.5352	
11	MATR3	SE	21	0.0116	0.0343	0.6899	
12	NONO	SE	29	0.0824	0.0858	1.0000	
13	PRPF8	SE	187	0.1812	0.2334	0.3280	
14	PUM1	SE	378	0.0960	0.1146	0.2789	
15	RBM15	SE	1050	0.0989	0.1279	0.1083	
16	SF3B4	SE	119	0.0948	0.1281	0.3870	
17	SMNDC1	SE	17	0.0936	0.2172	0.3609	
18	SRSF1	SE	671	0.0987	0.1352	0.0000	***
19	TARDBP	SE	114	0.1117	0.1335	0.4724	
20	TIA1	SE	76	0.0900	0.1219	0.9612	
21	TRA2A	SE	118	0.1002	0.1223	0.8093	
22	U2AF1	SE	186	0.0990	0.1442	0.2442	
23	U2AF2	SE	625	0.1005	0.1285	0.0795	.
24	UCHL5	SE	244	0.0955	0.1167	0.7666	
25	XRN2	SE	135	0.0914	0.1078	0.8046	
26	AQR	RI	865	0.2032	0.2011	0.9678	
27	CPSF6	RI	31	0.1585	0.1795	1.0000	
28	EFTUD2	RI	378	0.2356	0.2437	0.8578	
29	FXR1	RI	479	0.1730	0.2159	0.0210	*
30	HNRNPC	RI	314	0.1272	0.1609	0.4680	
31	HNRNPK	RI	33	0.1636	0.1741	0.9612	

Table S6: The active level of SURF-identified transcript sets in TCGA LAML and GTEx whole blood samples (continued).

	factor	event	size	AUC(GTEx)	AUC(TCGA)	adj. p-value	significance
32	HNRNPU	RI	59	0.1400	0.1544	1.0000	
33	IGF2BP2	RI	321	0.1668	0.1888	0.1623	
34	KHSRP	RI	38	0.1813	0.2356	0.9612	
35	LIN28B	RI	53	0.1300	0.1712	0.8046	
36	LSM11	RI	75	0.2315	0.2434	0.9400	
37	METAP2	RI	38	0.3286	0.3696	0.7725	
38	NCBP2	RI	111	0.1727	0.2010	0.4749	
39	PRPF8	RI	500	0.2050	0.2226	0.3788	
40	PUM1	RI	218	0.1578	0.1749	0.5352	
41	RBM15	RI	754	0.1986	0.2077	0.7666	
42	SF3B4	RI	362	0.2051	0.2322	0.4618	
43	SMNDC1	RI	68	0.2721	0.2790	1.0000	
44	SRSF1	RI	596	0.1648	0.1837	0.3956	
45	TARDBP	RI	137	0.1696	0.2124	0.5935	
46	TIA1	RI	122	0.1524	0.1816	0.9612	
47	TRA2A	RI	180	0.2059	0.2101	1.0000	
48	U2AF2	RI	354	0.2026	0.2193	0.8994	
49	UCHL5	RI	190	0.1880	0.2174	0.7725	
50	XRN2	RI	168	0.1696	0.1704	1.0000	
51	APOBEC3C	A3SS	42	0.2495	0.2693	0.8994	
52	AQR	A3SS	1167	0.2265	0.2625	0.0000	***
53	EFTUD2	A3SS	179	0.2383	0.2685	0.3870	
54	EIF3G	A3SS	38	0.1975	0.2643	0.8994	
55	FXR1	A3SS	563	0.1678	0.2276	0.0000	***
56	HNRNPC	A3SS	482	0.1513	0.1799	0.3788	
57	HNRNPK	A3SS	40	0.2216	0.1848	0.5054	
58	HNRNPU	A3SS	97	0.1862	0.2707	0.2442	
59	IGF2BP2	A3SS	261	0.1906	0.2154	0.2442	
60	KHSRP	A3SS	40	0.1532	0.3063	0.0266	*
61	LIN28B	A3SS	325	0.1470	0.1678	0.7725	
62	NCBP2	A3SS	23	0.1926	0.2266	0.9612	
63	NONO	A3SS	11	0.2721	0.0989	0.4680	
64	PRPF8	A3SS	297	0.2315	0.2574	0.4791	
65	PUM1	A3SS	348	0.1491	0.2060	0.0000	***
66	PUS1	A3SS	80	0.2267	0.2569	0.6655	
67	RBM15	A3SS	888	0.1885	0.2373	0.0000	***

Table S6: The active level of SURF-identified transcript sets in TCGA LAML and GTEx whole blood samples (continued).

	factor	event	size	AUC(GTEx)	AUC(TCGA)	adj. p-value	significance
68	RBM22	A3SS	12	0.2311	0.3195	0.5352	
69	SF3B4	A3SS	294	0.2108	0.2646	0.0000	***
70	SMNDC1	A3SS	60	0.2375	0.3135	0.3008	
71	SRSF1	A3SS	587	0.1663	0.2060	0.0000	***
72	TARDBP	A3SS	191	0.2384	0.2809	0.2408	
73	TIA1	A3SS	70	0.2025	0.2338	0.7321	
74	TRA2A	A3SS	181	0.1869	0.2003	0.7725	
75	U2AF1	A3SS	52	0.2194	0.2598	0.6877	
76	U2AF2	A3SS	621	0.1568	0.1915	0.0173	*
77	UCHL5	A3SS	231	0.1610	0.1910	0.1835	
78	XRN2	A3SS	167	0.2072	0.1952	0.8994	
79	APOBEC3C	A5SS	30	0.2198	0.3393	0.0795	.
80	AQR	A5SS	930	0.1914	0.2389	0.0000	***
81	EFTUD2	A5SS	59	0.2082	0.2213	0.9612	
82	FXR1	A5SS	425	0.1552	0.2146	0.0000	***
83	HNRNPC	A5SS	370	0.1312	0.1827	0.0131	*
84	HNRNPK	A5SS	21	0.1444	0.1744	0.8104	
85	HNRNPU	A5SS	61	0.1576	0.1661	1.0000	
86	IGF2BP2	A5SS	286	0.1543	0.1769	0.3076	
87	LIN28B	A5SS	216	0.1819	0.2068	0.7666	
88	NCBP2	A5SS	30	0.1739	0.2399	0.2373	
89	NONO	A5SS	12	0.1208	0.2069	0.4071	
90	PRPF8	A5SS	280	0.2093	0.2736	0.0000	***
91	PUM1	A5SS	239	0.1525	0.1870	0.1212	
92	RBM15	A5SS	688	0.1669	0.2158	0.0000	***
93	SF3B4	A5SS	120	0.2075	0.2385	0.3076	
94	SMNDC1	A5SS	19	0.3087	0.3689	0.9283	
95	SRSF1	A5SS	531	0.1547	0.1969	0.0000	***
96	TARDBP	A5SS	81	0.2150	0.2487	0.8233	
97	TIA1	A5SS	57	0.1302	0.2118	0.1475	
98	TRA2A	A5SS	166	0.1576	0.1940	0.3552	
99	U2AF2	A5SS	481	0.1488	0.2010	0.0028	**
100	UCHL5	A5SS	172	0.1226	0.1480	0.7666	
101	XRN2	A5SS	136	0.1655	0.1860	0.6306	
102	AQR	AFE	481	0.0671	0.0548	0.9612	
103	CPSF6	AFE	52	0.0392	0.0720	1.0000	

Table S6: The active level of SURF-identified transcript sets in TCGA LAML and GTEx whole blood samples (continued).

	factor	event	size	AUC(GTEx)	AUC(TCGA)	adj. p-value	significance
104	EFTUD2	AFE	93	0.1118	0.1156	1.0000	
105	FMR1	AFE	36	0.1047	0.1221	0.9678	
106	FXR1	AFE	625	0.0584	0.0638	1.0000	
107	HNRNPC	AFE	669	0.0649	0.0802	1.0000	
108	HNRNPU	AFE	81	0.1017	0.0924	0.9612	
109	IGF2BP2	AFE	683	0.0662	0.0672	1.0000	
110	LIN28B	AFE	692	0.0612	0.0693	1.0000	
111	LSM11	AFE	16	0.1784	0.1505	0.9678	
112	METAP2	AFE	48	0.1423	0.1173	0.9023	
113	NCBP2	AFE	96	0.0872	0.1228	0.4050	
114	NONO	AFE	69	0.1176	0.1686	0.5352	
115	PRPF8	AFE	213	0.0949	0.1029	1.0000	
116	PUM1	AFE	453	0.0532	0.0656	1.0000	
117	RBM15	AFE	1239	0.0584	0.0616	1.0000	
118	SF3B4	AFE	117	0.0710	0.0832	1.0000	
119	SMNDC1	AFE	26	0.2601	0.3352	0.9799	
120	SRSF1	AFE	689	0.0581	0.0679	1.0000	
121	TARDBP	AFE	278	0.0520	0.0690	1.0000	
122	TIA1	AFE	85	0.1104	0.1541	0.8994	
123	TRA2A	AFE	380	0.0827	0.0988	1.0000	
124	U2AF1	AFE	28	0.1222	0.1658	0.9530	
125	U2AF2	AFE	374	0.0662	0.0735	1.0000	
126	UCHL5	AFE	382	0.0813	0.0970	0.9612	
127	XRN2	AFE	244	0.1002	0.1193	0.8147	
128	AQR	A5U	144	0.0827	0.0600	0.3956	
129	EFTUD2	A5U	24	0.1208	0.1263	1.0000	
130	EIF3G	A5U	133	0.0944	0.1002	1.0000	
131	FMR1	A5U	66	0.1315	0.1239	1.0000	
132	FUS	A5U	61	0.1611	0.1997	1.0000	
133	FXR1	A5U	823	0.0869	0.1056	1.0000	
134	GEMIN5	A5U	335	0.1080	0.1219	0.9799	
135	GRWD1	A5U	243	0.0980	0.1078	1.0000	
136	HNRNPC	A5U	918	0.0659	0.0864	1.0000	
137	IGF2BP1	A5U	44	0.1583	0.2600	0.0507	.
138	IGF2BP2	A5U	1318	0.0768	0.0827	1.0000	
139	LIN28B	A5U	1113	0.0683	0.0786	1.0000	

Table S6: The active level of SURF-identified transcript sets in TCGA LAML and GTEx whole blood samples (continued).

	factor	event	size	AUC(GTEx)	AUC(TCGA)	adj. p-value	significance
140	METAP2	A5U	76	0.1074	0.1074	1.0000	
141	NCBP2	A5U	502	0.0882	0.0827	0.9612	
142	NONO	A5U	63	0.1192	0.1182	1.0000	
143	PUM1	A5U	1026	0.0818	0.0836	1.0000	
144	RBM15	A5U	1687	0.0541	0.0686	1.0000	
145	SERBP1	A5U	100	0.0869	0.0903	1.0000	
146	SF3B4	A5U	38	0.1020	0.0718	0.8233	
147	SND1	A5U	113	0.1456	0.2063	0.9612	
148	SRSF1	A5U	1295	0.0699	0.0850	0.5988	
149	SUPV3L1	A5U	132	0.0828	0.0774	1.0000	
150	TARDBP	A5U	184	0.0740	0.0848	0.9612	
151	TRA2A	A5U	796	0.0954	0.1123	0.8046	
152	U2AF2	A5U	401	0.0781	0.1005	0.9612	
153	UCHL5	A5U	885	0.0774	0.1032	0.5352	
154	XRN2	A5U	284	0.0726	0.0747	1.0000	
155	CPSF6	IAP	94	0.0592	0.0804	1.0000	
156	EIF3G	IAP	17	0.0000	0.0232	0.9612	
157	FXR1	IAP	587	0.0241	0.0451	0.8994	
158	HNRNPC	IAP	377	0.0280	0.0631	0.6306	
159	HNRNPU	IAP	38	0.0640	0.0574	1.0000	
160	IGF2BP2	IAP	642	0.0319	0.0350	0.9799	
161	LIN28B	IAP	803	0.0423	0.0524	1.0000	
162	LSM11	IAP	73	0.0748	0.0895	0.9612	
163	NCBP2	IAP	28	0.0595	0.0577	1.0000	
164	NONO	IAP	20	0.1810	0.1652	1.0000	
165	PRPF8	IAP	73	0.0676	0.1208	0.8994	
166	PUM1	IAP	182	0.0318	0.0433	0.8994	
167	RBM15	IAP	1009	0.0282	0.0506	0.9612	
168	SF3B4	IAP	129	0.0379	0.0565	0.6535	
169	SRSF1	IAP	552	0.0295	0.0515	0.2373	
170	TARDBP	IAP	196	0.0213	0.0533	0.9612	
171	TIA1	IAP	162	0.0376	0.0535	0.9612	
172	TRA2A	IAP	326	0.0281	0.0568	0.7441	
173	U2AF2	IAP	565	0.0377	0.0577	0.8093	
174	UCHL5	IAP	277	0.0554	0.0906	0.3870	
175	XRN2	IAP	82	0.0627	0.0815	0.9612	

Table S6: The active level of SURF-identified transcript sets in TCGA LAML and GTEx whole blood samples (continued).

	factor	event	size	AUC(GTEx)	AUC(TCGA)	adj. p-value	significance
176	APOBEC3C	TAP	39	0.1083	0.1875	0.4680	
177	AQR	TAP	589	0.0350	0.0516	0.1475	
178	CPSF6	TAP	194	0.0665	0.0877	1.0000	
179	EIF3G	TAP	110	0.0844	0.1276	0.8899	
180	FMR1	TAP	26	0.0975	0.1037	1.0000	
181	FUS	TAP	67	0.1635	0.2139	0.9400	
182	FXR1	TAP	709	0.0596	0.0771	1.0000	
183	GRWD1	TAP	365	0.0664	0.0968	0.3552	
184	HNRNPC	TAP	1941	0.0505	0.0680	0.9612	
185	HNRNPL	TAP	18	0.1735	0.1623	1.0000	
186	HNRNPU	TAP	184	0.1019	0.1360	0.9678	
187	IGF2BP1	TAP	53	0.0870	0.0647	0.8994	
188	IGF2BP2	TAP	1339	0.0644	0.0735	0.3280	
189	KHDRBS1	TAP	12	0.1244	0.1669	0.8994	
190	LIN28B	TAP	1792	0.0512	0.0683	0.9612	
191	METAP2	TAP	104	0.1598	0.1805	0.9307	
192	NCBP2	TAP	91	0.0563	0.0564	1.0000	
193	NONO	TAP	18	0.0945	0.0112	0.4050	
194	PHF6	TAP	14	0.1096	0.1047	1.0000	
195	PPIL4	TAP	32	0.0099	0.1065	0.8093	
196	PUM1	TAP	611	0.0514	0.0595	0.6877	
197	PUS1	TAP	102	0.0378	0.0544	0.9612	
198	RBM15	TAP	1563	0.0425	0.0650	0.6243	
199	SRSF1	TAP	582	0.0532	0.0566	0.9612	
200	TARDBP	TAP	160	0.0628	0.0908	0.6090	
201	TIA1	TAP	255	0.1114	0.1407	0.9678	
202	TRA2A	TAP	436	0.0580	0.0696	0.5935	
203	U2AF1	TAP	14	0.0634	0.1077	1.0000	
204	U2AF2	TAP	886	0.0528	0.0742	0.2746	
205	UCHL5	TAP	728	0.0574	0.0788	0.5352	
206	UPF1	TAP	111	0.1202	0.1000	0.8578	
207	XRN2	TAP	170	0.0609	0.0804	0.5935	

Table S7: Summary of the numbers (and the percentages) of retained genes, transcripts, and RNA-seq reads (in TPM), averaged across all samples. The filtering strategies (first column) are defined with isoform relative abundance (in percent, IP) or TPM. The first row presents the statistics for the complete set of genome annotation from GENCODE version 24. Rows 2-6 represent pre-filtering strategies with different thresholding settings. The percentages of retained genes, transcripts, and RNA-seq reads (in TPM) are listed in parentheses.

	#gene	#transcripts	Sum of TPM
Complete set	60,554	200,094	1,000,000
IP$\geq$ 5%	40,972 (67.7%)	147,963 (73.9%)	991,810.4 (99.2%)
IP$\geq$ 10%	40,972 (67.7%)	134,063 (67.0%)	980,396.0 (98.0%)
IP$\geq$ 25%	40,972 (67.7%)	108,601 (54.3%)	937,847.9 (93.8%)
TPM$\geq$ 1%	17,767 (29.3%)	82,535 (41.2%)	998,544.9 (99.9%)
IP$\geq$ 5% & TPM$\geq$ 1%	17,767 (29.3%)	74,733 (37.3%)	990,518.9 (99.1%)
