# Supplementary material for: SURF: integrative analysis of a compendium of RNA-seq and CLIP-seq datasets highlights complex governing of alternative transcriptional regulation by RNA-binding proteins
Source: Genome Biol. 2020 Jun 12;21:139. doi: 10.1186/s13059-020-02039-7 (PMC7291511; doi:10.1186/s13059-020-02039-7)
Supplement: Supplementary file 2 — Additional file 2 Review history [file 13059_2020_2039_MOESM2_ESM.docx]

Review History

**First round of review**

**Reviewer 1**

**Were you able to assess all statistics in the manuscript, including the appropriateness of statistical tests used?**

Yes: The methods generally seem sound; however some additional details would be helpful in describing the exact values going into some of the calculations (I note the AUC calculation in particular, which I think is described a bit confusingly)

**Were you able to directly test the methods?**

No

**Comments to author:**

The manuscript by Chen & Keles presents SURF, a combination of analysis approaches to both identify differential RNA processing sites from RNA-seq data (with DrSeq), and then perform deeper integration of CLIP and RNA-seq datasets to identify regulatory patterns controlled by RNA binding proteins. The DrSeq approach is interesting, and the SURF approach does seem to provide appealing benefits over the standard approaches to integrate CLIP and RNA-seq to identify governing principles of how RNA binding proteins differentially regulate their targets.

Major comments:

DrSeq: The DrSeq approach is quite interesting, but I would be interested in seeing a bit more comparison with existing methods than the TPR/FDR analysis in Fig 3a / S6. Most notably, the manuscript focuses on identifying significantly differential events but doesn't discuss the degree of inclusion or exclusion (percent spliced in / PSI) of the alternative event, which is typically the quantitation used for alternative RNA isoforms. I understand that the DrSeq question is more focused on identification of significant differential events than quantification of the difference, but I wonder if the DrSeq approach is better (or worse) at larger (or smaller) changes in isoform usage (delta-PSI) - in other words, is DrSeq more accurately identifying larger changes in splicing, or more sensitive at identifying events with smaller changes?

Similarly, as Fig. 3C is a re-analysis of alternative isoform usage from the ENCODE resource (which has rMATS splicing calls), it would be valuable to see a direct comparison of the # of events across methods for equivalent event types. I think it would also be helpful here to show & discuss discrete examples of stereotypical events that rMATS calls but DrSeq does not (and vice versa) to give a sense of why each method is showing either differential specificity or different types of false-positives.

Relatedly, the authors note that "SURF is implemented in R [29] and released as a Bioconductor [30] package surf.", but I don't see that package as publicly available yet, so the authors should confirm that 'analysis module 1' (DrSeq) is being made publicly available in that package (it would be helpful to see the documentation that is accompanying that R package to confirm what is being made available).

--

The mutation analysis in Fig. 6b is intriguing, but I think it would be stronger with a bit deeper comparison here of other more primitive ways to sub-select regions - e.g. how much higher are SURF-inferred features than, for example, only highly conserved regions within the linked features, or only regions significantly bound in eCLIP data? (also just a clarification note - is Fig. 6b using all 78k SURF-inferred or the 27k noted associated with 2+ RBPs?) I think this result is interesting, but I'm unclear how much of the benefit is really coming from the unique aspects of the SURF analysis.

--

I'm a bit confused about the analysis and result in Fig 6d. This may be just a writing clarification issue, but I'm unclear what exactly is going into the AUC calculation, and why the authors then conclude a shift towards shorter transcripts. In general, I think this section would be improved by expanding the methods section (or walking through the calculations a bit more in supplemental figures), particularly as the result is quite surprising

a)	For a class of ATR events and associated elements (say, TARDBP.A3SS) here, are both included- and excluded-associated events included in the same AUC calculation?

b)	I'm confused what the interpretation of the median AUC means - as above if both included- and excluded-associated elements are included, that seems to complicate the analysis. More generally though, I'm not clear what the value is that's being used (the methods says 'transcriptome expression across samples' - does that mean isoform-level expression (essentially a PSI-like value) or something else?

c)	I share the author's concern that this could represent a technical difference between the two datasets; in addition to the sub-setting analysis that they performed, it would be good to see some general summaries of the datasets - do the GTEx and TCGA data in general have the same average read lengths & RNA quality? Is it possible that the observation is due to just a mismatch in cell-types (whole blood versus AML)?

d)	The global shift seen across many RBPs suggests an extremely broad conclusion (that AML generally shows decreased transcript size / increased exclusion of alternative events) - can this be reproduced using other analysis approaches? Similar findings have been made for alternative 3'UTR length in cancers, but I don't recall this degree of broad shifting in isoform usage has been noted previously.

---

My other major comment is that I would recommend the language be more careful in interpretation of the directionality for eCLIP and RNA-seq overlaps, particularly for spliceosomal components (in particular AQR, EFTUD2, SF3B4) - e.g.

Focusing on SE events, for instance, we observed that AQR and EFTUD2 binding at the flanking intronic regions of event body (location features γ and ζ) associated with differential SE significantly and promoted both exclusion and inclusion (Supplementary Figures S13, S14, and S15).

Trivially, these RBPs (as core components of the major spliceosome) presumably bind to every spliced intron in the transcriptome at some level; thus, I think it remains an open question whether strong (and/or differential) enrichment in eCLIP of these spliceosomal comments reflects associations that promote or drive alternative splicing regulation, or whether they are more appropriately considered as a readout of spliceosomal dwell time and isoform usage. In other words, whether high AQR enrichment at an intron reflects more spliceosome activity or instead reflects longer spliceosome occupancy due to slower splicing progress at that site (which would give opposing hypotheses on what the observed concordance between knockdown RNA-seq and eCLIP means). Indeed, this may explain the observation that they promote both exclusion and inclusion - that increased spliceosomal RBP 'binding' is really reflecting slower splicing at weaker introns, which are then more likely differentially effected upon knockdown of spliceosomal components.

Minor comments

In the methods section "Generation of feature signals from eCLIP-seq data", could the authors clarify "Finally, the difference between normalized IP and input samples is reported as feature signal. " - is this difference the subtracted difference or some sort of normalized fold-change?

I was able to access the web resource at http://www.statlab.wisc.edu/shiny/surf/ , but the https:// link provided in the manuscript wasn't functional when I accessed it

**Reviewer 2**

**Were you able to assess all statistics in the manuscript, including the appropriateness of statistical tests used?**

Yes: The statistical methods are appropriate.

**Were you able to directly test the methods?**

Yes

**Comments to author:**

This manuscript reports a new analysis method, SURF, that integrates multiple types of data to analyze patterns of alternative RNA isoforms, and their regulation by RNA-binding proteins. Compared to existing methods, SURF showed superior performance. In addition, it offers a comprehensive set of modules with utilities that are not afforded by other methods. The integration of CLIP and RNA-seq data is a powerful means to infer regulatory mechanisms of RNA processing. This work addresses significant challenges in studying RNA regulation, and the tools described here will be welcomed additions to the field. I have only a few comments to hopefully help to improve the manuscript.

MAJIQ (modeling alternative junction inclusion quantification) is another tool that analyzes alternative isoforms using local events, and can handle replicates. The authors should compare their method to this method, if possible.

With the locations features identified for the target exons of each RBP, it would be good to carry out motif analysis, which can then be compared to known motifs of the RBP. This analysis may also reveal novel motifs.

In the analysis of somatic mutations within location features, are there any mutations that may alter RBP binding (e.g., allele-specific binding or creation/loss of binding of certain RBPs)?

A powerful application of SURF is comparing activity of RBPs across data sets or conditions. In their application, the authors analyzed GTEx and TCGA data. Could the authors clarify how batch-related effects were handled? Did the usage of the described control sets eliminate batch effects completely?

**Reviewer 3**

**Were you able to assess all statistics in the manuscript, including the appropriateness of statistical tests used?**

No

**Were you able to directly test the methods?**

No

**Comments to author:**

This is a very strong and well written manuscript integrating RNA-seq and CLIP-seq data to comprehensively study alternative splicing events, including exon skipping, alternative 3' or 5' splicing, intron retention, alternative first exon or alternative 5'UTR usage, as well as intronic or tandem alternative polyadenylation.

The strength of the manuscript lies in the integration of RNA-seq (including knockdown of RBPs to verify splicing events) with CLIP-seq data to comprehensively identify the regions of RNA in which these RBPs bind and their impact at the transcript level.

As I am not a computational biologist, I cannot comment on the robustness of the bioinformatic analysis, but the biology/analysis part of the manuscript is well done and provides a number of new biological insights, and should be of broad use to the field.

I have one or two questions regarding the final part of the analysis in their manuscript, namely their integration with TCGA data that the authors should resolve before publication.

1. What is the mutational status of their TCGA leukaemia samples? For example, do they have mutations in splicing factors such as SF3B1, U2AF1, PRPF8, SRSF2, that are commonly observed in leukaemias?

2. How does this affect their findings and the transcripts whose expression is changed in TCGA samples compared to whole blood samples?

**Authors Response**

**Point-by-point responses to the reviewers’ comments:**

*We thank the reviewers for their careful reading of our manuscript and constructive comments. We outline below our point-by-point response to each of the reviewer’s comments. For the ease of reading, we itemized all points raised in the order that they were brought up and addressed them one by one. Furthermore, we also indicated how and where the text was modified for each response. Overall, the key points of this revision can be summarized as:*

*1.	Additional comparisons of DrSeq with rMATS and MAJIQ, which further highlighted advan-tages of DrSeq in detecting differential ATR events.*

*2.	An overall comparison of analysis of ENCODE compendium with rMATS and DrSeq, which recapitulated the differences we have seen between rMats and DrSeq in our simulation exper-iments.*

*3.	De novo sequence analysis of SURF-inferred location features, which captured several known RBP binding motifs and revealed motif multi-modality for some RBPs.*

*4.	Refinement of integration of results with external transcriptome resources (TCGA and GTEx), which confirmed widely observed shortening of 3' UTRs during tumor genesis and cancer pro¬gression and revealed widespread contribution of differential regulation of alternative splicing (as opposed to alternative first exon or alternative 5' UTR).*

*We believe these results further strengthened and highlighted the merits of SURF for studying RBPs through the lens of alternative transcriptional regulation.*

R1 Response to Reviewer #1 (R1)

R1.C1. “...Most notably, the manuscript focuses on identifying significantly differential events but doesn’t discuss the degree of inclusion or exclusion (percent spliced in / PSI) of the alternative event, which is typically the quantitation used for alternative RNA isoforms. I understand that the DrSeq question is more focused on identification of significant differential events than quantification of the difference, but I wonder if the DrSeq approach is better (or worse) at larger (or smaller) changes in isoform usage (delta-PSI) - in other words, is DrSeq more accurately identifying larger changes in splicing, or more sensitive at identifying events with smaller changes?”

*Response Figure R1: Comparison of the performances of DrSeq, rMATS, MAJIQ (under two settings), and two simple strategies for stitching DEXSeq inferences into ATR event level (DEXSeq-L and DEXSeq-C) under different signal-to-noise levels. See Supplementary Figure S11 of the manuscript for a detailed description.*

*Response: We agree with the reviewer that it would indeed be useful to look at the comparison results from the point of isoform usage. To address this, we stratified the simulated transcriptome data into three groups, where the changes in isoform usages (IsoPct as quantified by RSEM) are small, moderate, and large. Figure R1 (equivalently Supplementary Figure S11 of the manuscript) displays performances of different methods within each of these groups and indicates that DrSeq outperforms rMATS under all the three signal-to-noise settings in terms of true positive and false discovery rates. We added to these comparisons a more recent tool MAJIQ (recommended by Reviewer #2) and showed that DrSeq has favorable performance compared to this approach as well.*

*We added this result as a Supplementary Figure and referred to it in the main text.*

*(Page 4 paragraph 2) ... Higher empirical FDR for rMATS (under various signal-to-noise levels, Sup¬plementary Figure S11) can be attributed to ...*

R1.C2. “...it would be valuable to see a direct comparison of the # of events across methods for equivalent event types. I think it would also be helpful here to show& discuss discrete examples of stereotypical events that rMATS calls but DrSeq does not (and vice versa) to give a sense of why each method is showing either differential specificity or different types of false-positives.”

*Response: To address this, we compared the numbers of events detected by rMATS and DrSeq in both simulated and ENCODE data. While DrSeq and rMATS detected similar total numbers of differential AS events in simulations (Figure R2 or equivalently Supplementary Figure S9 of the manuscript), we observed differences in the numbers of detected SE, RI, A3SS, and A5SS events in the analysis of 104 shRNA-seq datasets. Specifically, using the rMATS results from the ENCODE portal, we observed that DrSeq detected fewer differential SE events and more differential RI, A3SS, and A5SS events than rMATS (Figure R3 or equivalently Supplementary Figure S13). This aligned well with the observations from simulated data, where rMATS detected 50.7% more differential SE events than DrSeq, albeit at a higher observed FDR, and considerably fewer differential A3SS (by 77.4%) and A5SS (by 89.2%) events. While evaluating the false discoveries of the two methods in this large-scale application is prohibitive due to lack of ground truth, the fact that real data analysis has similar overall tendencies as the simulated data is reassuring. Furthermore, we compared DrSeq and rMATS quantifications of differential event usage (Figure R4) in our simulations. While most of DrSeq false positives tended to concentrate at lower estimatedβEC*

*iρ(j)l (DrSeq parameter quantifying*

*differential event usage) and lower L(PSI) values, false positives of rMATS showed markedly larger variation in terms of quantified effect sizes (both with estimatedβEC*

*iρ(j)l and L(PSI)). This likely reflects how the use of all reads relevant for the gene by DrSeq improves REU quantification. We further provided a genome browser display (Supplementary Figure S12) of an example with detailed data tables (Supplementary Tables S1 and S2), where an SE event was falsely identified as differentially regulated by rMATS but not by DrSeq.*

*We added these new results and the relevant discussion to Supplementary Figures and referred them in the main text as follows. For simulated data:*

*(Page 4 paragraph 2) ... all count-based methods (DrSeq and two variants of DEXSeq) achieve sig¬nificantly higher TPR and lower FDR, compared to rMATS, despite detecting comparable number of events (Supplementary Figure S9)...*

*For the false positive example of rMATS:*

*(Page 4 paragraph 2) ... rMATS only utilizes the reads nearby the event body and splice junctions, in contrast to the other count-based methods which account for all reads relevant to the genes (Supple¬mentary Figure S12 and Supplementary Tables S1 and S2)...*

*For real data:*

*(Page 4 paragraph 5) ... we observed that a higher level of transcriptional complexity resulted from AS in the human genome than previously revealed by rMATS (Supplementary Figure S13) ....*

*Response Figure R2: Comparison of the number of detected differential ATR events by various event-based methods in human transcriptome-based simulations. See Supplementary Figure S9 of the manuscript for a detailed description.*

R1.C3. “..., but I don’t see that package as publicly available yet, so the authors should confirm that ’analysis module 1’ (DrSeq) is being made publicly available in that package (it would be helpful to see the documentation that is accompanying that R package to confirm what is being made available).”

*Response Figure R3: Comparison of differential AS detection by DrSeq and rMATS in 104 short-hairpin RNA knock-down followed by RNA-seq (with paired control) experiments in K562 cells. See Supplementary Figure S13 of the manuscript for a detailed description.*

*Response Figure R4: Comparison of false positives of DrSeq and rMATS in human transcriptome-based simulations. Each point depicts the log_2_ REU fold change estimated by DrSeq and the difference in inclusion level (ΔPSI) estimated by rMATS for one falsely detected SE event, among the 11, 536 SE events commonly tested by both methods. The panel top strips indicate the method by which the false positive differential SE is identified with the numbers of false positives paren­theses.*

*Response: The package, surf, is publicly available at https://github.com/keleslab/surf, and we are in the final stages of Bioconductor submission. As the reviewer might appreciate, the bar to Bioconductor contributions is significantly higher than regular CRAN releases. Our group has a proven record of contributing our work to Bioconductor (e.g., mosaics, ChIPexoQual, pram and atSNP), and surf will also appear shortly.*

R1.C4. “...The mutation analysis in Fig. 6b is intriguing, but I think it would be stronger with a bit deeper comparison here of other more primitive ways to sub-select regions - e.g. how much higher are SURF-inferred features than, for example, only highly conserved regions within the linked features, or only regions significantly bound in eCLIP data? (also just a clarification note - is Fig. 6b using all 78k SURF-inferred or the 27k noted associated with 2+ RBPs?) I think this result is interesting, but I’m unclear how much of the benefit is really coming from the unique aspects of the SURF analysis.”

*Response: We appreciate the reviewer’s suggestions on finding additional genomic regions for which we can quantify mutation enrichment. Indeed, although the control sets we constructed form a nested series, it can be beneficial to examine some alternatives. To check one possibility (among many), we quantified the mutation enrichment in regions significantly bound in eCLIP-seq data (peaks with IDR < 0.01). Specifically, we collected (i) peaks of 104 RBPs studied in analysis module 1 and (ii) peaks of 54 RBPs for which we identified at least one functional association in analysis module 2. The two collections of peaks cover a total length of (i) 30.77 million bp and (ii) 24.16 million bp, respectively. Using the mutation data from ICGC projects, we observed an enrichment level of 17.08 MPK in (i) and 17.00 MPK in (ii) respectively. Both measures of mutation enrichment are higher than “gene” but lower than “span” (Figure 6ab of the manuscript and significantly lower than that of the SURF-inferred location features). These comparisons suggest that the 71,667 SURF-inferred location features do collectively offer a set of genomic regions that are potentially more relevant in terms of mutational data than solely using CLIP-seq data. We have included this additional analysis in section “SURF-identified location features are enriched for somatic mutations from ICGC and TCGA”.*

R1.C5. “...This may be just a writing clarification issue, but I’m unclear what exactly is going into the AUC calculation...

*Response: We thank the reviewer for these clarification questions about the AUC calculations. We updated this section to clarify the calculations and provide further supporting data. We started by providing another illustrative Figure R5 (equivalently Supplementary Figure S6 of the manuscript) to demonstrate the idea of area under recovery curve (AUC): (a) Given a target ge-nomic feature set (e.g., gene set or transcript set) and a descending ordering of all the features (e.g., ranked genes based on gene expression), the recovery curve (solid line) depicts the number of target genomic features among the top-ranked genomic features. For example, the set of top 510 features (marked by the vertical blue dashed line) harbors 40 target features. (b) Recovery curve in percentage scale. Both axes are re-scaled to [0, 1]. The blue area under the recovery curve (AUC)*

*quantifies the activity level of target genomic set.*

*We added this information to the Supplementary Figure and referred to it in the main text.*

*(Page 10 paragraph 5) ... The AUC evaluates the ranking of its elements compared to other quantified units in the sample in terms of expression (Supplementary Figure S6)...*

*Response Figure R5: Illustration of AUC calculation in discovery module of SURF. See Supple­mentary Figure S6 of the manuscript for a detailed description.*

R1.C6. “...a) For a class of ATR events and associated elements (say, TARDBP.A3SS) here, are both included- and excluded-associated events included in the same AUC calculation?...”

*Response: Since the AUC-based discovery module of SURF lifts the SURF-inferred location features to transcript (or gene) level to define a target set for an RBP (i.e., interaction of the RBP with these SURF-inferred location features contribute to existence of these transcripts), we merged both inclusion- and exclusion-associated events. This is further motivated by the empirical observation that many transcripts will harbor both significant inclusion and exclusion events and, thereby, the location features associated with them.*

R1.C7. “...b) I’m confused what the interpretation of the median AUC means - as above if both included- and excluded-associated elements are included, that seems to complicate the analysis. More generally though, I’m not clear what the value is that’s being used (the methods says ’transcriptome expression across samples’ - does that mean isoform-level expression (essentially a PSI-like value) or something else?...”

*Response: We apologize for not making these concepts more clear. We put in extra effort to clarify them. In summary, SURF-identified location features are regions in the vicinity of differential ATR events. They associate with the differential ATR status of the event and harbor eCLIP-seq signal for the RBP. Therefore, they enable us to define target gene sets and transcript sets for a given RBP. Regardless of whether the location feature is identified by association with inclusion or exclusion event, its makes the gene and the corresponding sets of transcripts that it is a part of a target for RBP. Therefore, all the AUC calculations are either at the gene or the transcript level and do not have any further inclusion exclusion relevance, and are based on TPM values. In the revision, we have taken advantage of isoform percentage quantification of transcripts. Isoform percentage of a transcript depicts the percentage of its abundance over its parent gene’s abundance. Comparison of isoform percentage served as an alternative to AUC measures and supported similar conclusions as we outline below in our response to R1.C9.*

R1.C8. “...c) in addition to the sub-setting analysis that they performed, it would be good to see some general summaries of the datasets - do the GTEx and TCGA data in general have the same average read lengths & RNA quality? Is it possible that the observation is due to just a mismatch in cell-types (whole blood versus AML)?...”

*Response: We have indeed scrutinized these datasets further. The GTEx and TCGA com¬pendium data were recomputed with a universal pipeline by UCSC Toil with uniform quality control [1]. This resulted in expression estimates for ∼ 60, 000 genes and ∼ 200, 000 transcripts, including many LncRNAs [2]. While we have not noticed obvious differences in data quality from the point of standard metrics, our analysis did reveal that the TCGA samples tend to have more detectable genes as loosely defined by TPM > 0.001. In other to dampen the impact of unforeseen unmeasured confounding, we designed additional controls by further matching the control sets to their target counterparts in their overall AUC as we outline in our response to the next point, R1.C9.*

R1.C9. “...d) The global shift seen across many RBPs suggests an extremely broad conclusion (that AML generally shows decreased transcript size / increased exclusion of alternative events) - can this be reproduced using other analysis approaches? Similar findings have been made for alternative 3’UTR length in cancers, but I don’t recall this degree of broad shifting in isoform usage has been noted previously...”

*Response: Thank you for raising this point. This motivated us to compare target sets of RBPs not only in terms of AUCs but also with isoform percentages, which reflects the usage of a transcript in comparison to other transcripts of the same gene, as an independent metric. In summary, all the additional controls we have used and the metrics we utilized led to similar conclusions as before. We restructured Figure 6 of the manuscript (see Figure R6 below) to make it more accessible. The overall flow is now as follows: (1) We first lifted SURF-inferred feature sets to gene level and defined gene targets of RBPs. We then computed activity levels of these gene sets by AUC in both GTEx and TCGA samples. Next, we constructed control sets for each RBP matching its target numbers of genes and also the mean AUC of its target genes. Figure 6c of the manuscript displays the mean AUCs of these genes sets (one target and one control set for each RBP) in TCGA versus GTEx and confirms that targets of RBPs do indeed show a shift in activity level in TCGA samples compared to GTEx samples. In contrast, the control sets, matching both in size and the average AUC to the target sets, do no exhibit such a shift. This solidifies that the overall shift is unlikely an artifact of unadjusted effects. (2) Next, we further dissected this result by considering transcript level targets of RBPs (each target gene will contribute one or more transcripts to the transcript-level target set) and constructing control transcript sets from the transcripts of the same gene that are not in the target set. This is also controls for the overall expression of the gene that the transcripts originate from. Next, we quantified activities of the target and control transcript sets with a metric that does not rely on AUC by utilizing isoform percentage (IsoPct reported by RSEM) and evaluated change in isoform percentages between TCGA and GTEx samples. Figure 6d of the manuscript stratifies the results as a function of event types and also displays control sets built from the transcripts of the same gene that are not part of the event-specific targets sets of RBPs. Overall, transcript sets associated with AS events (SE, RI, A3SS, and A5SS) exhibited reduced isoform percentage. In contrast, transcript sets associated with APA (IAP and TAP) showed increased isoform percentage. This observation held true even when we deduplicated all RBP-event specific target sets by ensuring that target transcripts harboring multiple event types were excluded from the analysis (Figure R7 or equivalently Supplementary Figure S42 of the manuscript). This observation recapitulates the observation that shortening of 3' UTRs is widely observed in cancer and highlights that AS and*

*APA events contribute more to transcriptome diversity in cancer compared to ATI events.*

*We reorganized the whole section titled “SURF-identified ATR targets of RBPs exhibit dif¬ferential transcriptional activity between TCGA acute myeloid leukemia tumor and GTEx whole blood samples” around the above discussion as follows.*

*(Page 6 paragraph 4) We next queried whether specific transcript targets of RBPs behaved differentially among all isoforms originating from the same gene, across tissue conditions. To this end, for every transcript, we quantified the changes in isoform percentage (IsoPct as reported by RSEM) from GTEx whole blood samples to TCGA LAML samples. Isoform percentage of a transcript depicts the percentage of its abundance over its parent gene’s abundance and its comparison across conditions elucidates how the contribution of a transcript to overall gene abundance is changing across conditions. Comparison of isoform percentages revealed a diverse pattern across ATR event types (Figure 6d), suggesting a refined transcript level biological relevance of SURF-identified RBP targets. Specifically, transcript sets associated with AS events (AS, RI, A3SS, and A5SS) exhibited reduced isoform percentage (as compared to matched random control sets from other isoforms originating from the same gene) between normal (GTEx) and tumor (TCGA) tissues (Supplementary Figure S43). In contrast, transcript sets associated with APA events (namely IAP and TAP) exhibited increased isoform percentage in tumor samples compared to normal samples (e.g., SRSF1 in IAP, Figure 6e), indicating that transcripts harboring these event types contributed more to their parent gene’s overall abundance. This observation is further supported by multiple replicates of random control and after de-duplicating transcripts across event types (Supplementary Figures S41 and S42). Since ATR events are defined by variable bins, which originate from shorter transcripts (i.e., those with absent exonic parts), this observation suggests that the transcripts that terminate earlier at 3' end, as well as those with more internal “spliced-in” exonic parts, tend to be more transcriptionally active in the LAML cancer tissue compared to normal blood tissue. Shortening of 3' UTRs is widely observed during tumorigenesis and cancer progression [3, 4]. In addition to supporting this widely observed phenomenon, our results suggest that AS and APA events contribute more to transcriptome diversity in cancer compared to ATI events. To test the differential activity for individual transcript sets, we applied the discovery module of SURF, with their paired controls by including transcripts originating from the same corresponding target genes. Of all the RBP-ATR event type combinations, 18 of them (out of 131 with more than 100 transcripts) exhibited significantly different transcriptional activity between the whole blood (GTEx) samples and LAML (TCGA) samples, at FDR cut-off of 0.05 (Figure 6f). The detected transcript sets originate from 9 RBPs, among which FXR1, SRSF1, and U2AF2 associate with the most diverse ATR event types (SE, A3SS, A5SS for all three RBPs, and RI for FXR1 additionally), followed by AQR and HNRNPC through two event types (Supplementary Tables S7), many of which have previously reported implications in cancer (e.g., [5, 6]). All but two of these RBPs (FXR1 and PUM1) are splicing factor genes and U2AF1 exhibited significant driver mutation patterns across a wide variety of cancer types [21].*

*Response Figure R6: Integration of SURF results with large-scale datasets from ICGC, TCGA, and GTEx. See Figure 6 of the manuscript for a detailed description.*

*Response Figure R7: Comparison of ΔIsoPct for a subset of SURF-inferred target transcripts that uniquely associated with only one ATR event type. See Supplementary Figure S42 of the manuscript for a detailed description.*

R1.C10. “...My other major comment is that I would recommend the language be more careful in interpretation of the directionality for eCLIP and RNA-seq overlaps, particularly for spliceosomal components (in particular AQR, EFTUD2, SF3B4) - e.g....”

*Response: We appreciate the reviewer’s very helpful recommendation on the language. We made the appropriate changes to emphasize that analysis module 2 of SURF associates the inclu-sion/exclusion of ATR events directly with the eCLIP-seq signal itself and may not immediately extend to inferring dual roles for RBPs.*

*Specifically, we included the following remark when we describe interpretation of the differential ATR output.*

*(Page 3 paragraph 1) As a result, “increased REU upon RBP knockdown” indicates the exclusion of variable sub-exons under the wild-type condition. Similarly, a “decreased REU upon RBP knockdown” indicates that RBP tends to promote inclusion of variable sub-exons under the wild-type condition. We remark that this interpretation does not necessarily extend to core components of the spliceosome since these components are likely to interact with every spliced intron at some level regardless of the inclusion or exclusion outcome.*

R1.C11. “...could the authors clarify ‘Finally, the difference between normalized IP and input samples is reported as feature signal.’ – is this difference the subtracted difference or some sort of normalized fold-change?...”

*Response: This difference is the arithmetic difference of normalized signals of IP and input samples. Both the IP and input samples are processed with a standard procedure that takes into account the depth of the samples and the lengths of the genomic features (i.e., akin to FPKM), and this difference operation corresponds to “background correction” for the measurements from the IP samples.*

R1.C12. “I was able to access the web resource at http://www.statlab.wisc.edu/shiny/surf/ , but the https:// link provided in the manuscript wasn’t functional when I accessed it.”

*Response: We thank the reviewer for spotting this typo. The shiny app is host through de-partment server which only provides http:// access for shiny app. We removed the “s” from the URL.*

R2 Response to Reviewer #2

R2.C1. “MAJIQ (modeling alternative junction inclusion quantification) is another tool that ana-lyzes alternative isoforms using local events, and can handle replicates. The authors should compare their method to this method, if possible.”

*Response: Thank you for bringing MAJIQ to our attention. We have now included MAJIQ in our comparisons (Figure R1 or equivalently Supplementary Figure S8 of the manuscript and Supple¬mentary Figure S11 for additional stratification as a function of signal-to-noise ratio). Specifically, we considered two versions: one with default parameters and another with a more liberal param¬eter setting. While we observed that DrSeq has significantly better power than MAJIQ in our computational experiments from [7] which provide a fair setting for comparing different methods, a number of key differences between the event-based DrSeq, rMATS and the MAJIQ framework exist. First, MAJIQ does not generate summary statistics that can be leveraged for FDR control. Therefore, instead of targeting a particular FDR level as we have done for DrSeq and rMATS, we considered default and liberal parameter settings for MAJIQ and assessed empirical FDR. Sec¬ond, and more importantly, the experimental units of MAJIQ are local splice variations (LSVs) which are not based on predefined alternative regulation events and somewhat harder to interpret for downstream analysis. While MAJIQ provides annotations of LSVs into ATR event types, it predominantly reports SE, A3SS, and A5SS event types and includes a large degree of overlap among these types (Figure R8 or equivalently Supplementary Figure S7 of the manuscript), which prohibited direct comparison of the results at the ATR event level.*

*Response Figure R8: Annotation of local splice variations detected by MAJIQ on simulated RNA-seq data into ATR event types. See Supplementary Figure S7 of the manuscript for a detailed description.*

*We have discussed the scope of our comparisons with MAJIQ in the manuscript and included the relevant data.*

*(Page 3 paragraph 4) ... rMATS detects differential ATR for individual alternative splicing events as opposed to the differential exon usage in DEXSeq or the local splice variations in MAJIQ (Supplementary Figure S7)...*

R2.C2. “...With the locations features identified for the target exons of each RBP, it would be good to carry out motif analysis, which can then be compared to known motifs of the RBP. This analysis may also reveal novel motifs...”

*Response: This was an excellent suggestion and a direction we were already working on. We carried out a detailed de novo motif analysis on SURF-identified location feature. While this analysis recapitulated many known motifs of RBPs, it also revealed that sequence specificities of some RBPs exhibit multi-modality depending on the type of ATR event that the RBP is regulating. Specifically, 19 RBPs exhibited motif multi-modality of varying degrees which we quantified by the maximum and minimum Kullback-Leibler divergence between all the learnt PWMs of RBPs. We believe the motif analysis further highlights the advantages of studying RBPs through the lens of ATR events as SURF does.*

*We added the following new sub-section entitled “SURF-identified location features carry ATR- specific sequence motifs” to the paper.*

*(Page 5 paragraph 3) The analysis module 2 of SURF identified 34,795 differential ATR events associated with binding of at least one of 54 RBPs. Aggregation across these associations resulted in 71,667 SURF-inferred location features (FDR of 0.05 and feature signal cut-off of 20, Supplementary Methods), among which 34.9% (25,006) are associated with two or more RBPs. The numbers of inferred location features exhibited considerable heterogeneity across ATR types, with TAP having the largest numbers of inferred location features (19.2% and constituting 10.2% of all the possible location features of TAP event type), followed by A5U (15.5% and constituting 7.9% of all the possible location features of TAP event type) and SE (13.3% and constituting 5.5% of all the possible location features of A5U event type) classes (Supplementary Figure S30). Supplementary Figure S31 displays the distribution of genomic coverage by inferred features for all the eight event types.*

*(Page 5 paragraph 3) Recent machine learning applications to predict pre-mRNA processing highlighted strong predictive power of sequence variants [8, 9, 10]. While the SURF framework does not emphasize in silico prediction of ATR events from sequence input, we expected some location features to harbor sequence motifs for RBPs. Leveraging the ability of SURF to identify distinct location features for different ATRs regulated by the same RBP, we asked whether these location features harbored distinct sequence motifs. Specifically, we performed de novo motif analysis with the MEME suite [11] for location features of 222 RBP-ATR event type combinations involving 52 distinct RBPs (Materials and Methods). The analysis captured a number of known, experimentally validated RBP motifs [12], e.g., TARDBP, U2AF2, HNRNPC, and TIA1. We next clustered these RBP-event type combinations, which involved 30 RBPs for which significant motifs were identified, based on the similarities of their identified motifs (E-values ≤ 0.01) (Figure 5c). The event type-based clustering evident in Figure 5c is partly driven by the shared location features of RBPs for the same event type (Supplementary Figure S32). However, the extent of overlap between the location features of different RBPs for the same event type is relatively low (Supplementary Figure S33) and indicates marked contribution of sequence similarities of the RBP-event type-specific motifs to the clustering structure. Overall, de novo sequence analysis of SURF-inferred features unveiled multiple unique sequence motifs of RBPs for distinct ATR events. Specifically, 19 RBPs exhibited motif multi-modality of varying degrees which we quantified by the maximum and minimum Kullback-Leibler divergence between all the learnt PWMs of of individual RBPs (Supplementary Figure S34). For example, we observed that HNRNPC, a known poly(U)-binding regulator for the TAP class (E-value 3.0 × 10−68, Figure 5d) [13, 14, 15], potentially recognizes a 5'-CCUC-3' motif for the SE class (E-value 3.2 × 10−152, Figure 5e). In addition, this clustering reconfirmed that while TIA1 overall prefers uridine-rich sequences [16, 17, 18], it also interacts with an AU-riched sequences [19], 5'-AAUAAA-3', in event types IAP and TAP (Supplementary Figure S35).*

R2.C3. “...In the analysis of somatic mutations within location features, are there any muta-tions that may alter RBP binding (e.g., allele-specific binding or creation/loss of binding of certain RBPs)?...”

*Response: This is a question we are intimately interested in. In fact, in another work, we have developed a rigorous framework for evaluating in silico impact of SNPs on TF binding and evaluated all the dbSNP SNPs against ENCODE and JASPAR PWMs (results accessible via web application atsnp biostat wisc edu). Recently, Yang et al. [20] observed and identified wide-spread allele-specific binding (ASB) events in eCLIP-Seq data. Therefore, it is reasonable to expect a subset of the somatic mutations to potentially alter RBP binding. In an ongoing project, we are evaluating the in silico impact of SNPs and somatic mutations on RBP binding and its implications for TCGA datasets. We have chosen to mention this potential analysis in the Discussion.*

R2.C4. “...clarify how batch-related effects were handled...”

*Response: Our two key strategies for handling batch effects are (i) using metrics such as AUC that rely on rankings within a dataset rather than actual quantitative values (e.g., gene or transcript expression) and are therefore directly comparable across datasets of different cohorts; (ii) for each target set (either set of genes or transcripts) to be evaluated across TCGA and GTEx, generating matching control sets so that observations on the target set can be compared with observations on the control set. Overall, we refrained from directly comparing measurements from GTEx and TCGA samples. Specifically, results in Figures 6c, d, e, and f all rely on carefully matched control sets to adjust for unforeseen confounding factors. Reviewer #1 also posed a related question. We refer to R1.C7 and R1.C8 for further discussion.*

R3 Response to Reviewer #3

*We are happy to hear that the reviewer views our work to be of broad use to the field.*

R3.C1. “What is the mutational status of their TCGA leukaemia samples? For example, do they have mutations in splicing factors such as SF3B1, U2AF1, PRPF8, SRSF2, that are commonly observed in leukaemias?”

*Response: Somatic mutational landscape of splicing factors across TCGA samples has been stud¬ied in [21], where they documented 119 splicing factor genes that carry putative driver mutations in one or more cancer types. Specifically, three of the four splicing genes that the reviewer points out have putative driver mutations in the TCGA samples and have been selected as part of the Seiler [21] 119 splicing gene set (SF3B1, U2AF1, SRSF2). In the cohort of 173 TCGA LAML sam¬ples, we observed 2,544 somatic mutations (Table R1) residing within 1,876 genes. Specifically, we observed 2 missense mutations in the PRPF8 gene, and one missense mutation in SF3B1, and one in-frame deletion in SRSF2, and as many as 8 missense mutations in the U2AF1 gene. Leveraging this resource and the TCGA LAML samples, we checked the mutational status of the RBPs that contributed at least one significant differential transcript set in the module 3 of SURF: namely, AQR, FXR1, HNRNPC, PRPF8, PUM1, RBM15, SF3B4, SRSF1, and U2AF2. In this set, all but PUM1 and FXR1 RBPs are splicing factor genes. We observed that most of these genes do not have somatic mutations, except for PRPF8 and U2AF2. Of these, only U2AF2 made it to the list of 119 splicing factor genes with putative driver mutation. This observation further highlights one of the advantages of the SURF framework, that is, while the RBPs themselves may not have any coding mutations, their ATR targets could harbor mutations that might contribute to differential activity.*

R3.C2. “...How does this affect their findings and the transcripts whose expression is changed in TCGA samples compared to whole blood samples?...”

*Response: We thank the reviewer for bringing up this discussion point. The fact that all but two of the RBPs, which exhibited differential target activity, do not have somatic mutations does not effect the interpretation of our results and, in fact, highlights how SURF results can complement existing driver mutation analysis of TCGA data (e.g., from [21]). The discovery module of SURF delineates targets for a given RBP based on the results of module 2. These targets harbor ATR events that are differentially regulated by the RBP and harbor genomic regions eCLIP-seq signals of which directly associate with the differential ATR status of events. Therefore, these are likely direct targets of RBPs. These targets, defined completely independent of GTEx and TCGA data, may be showing differential activity in these samples due to multiple reasons. Two obvious ones are as follows. First, the RBP might be differentially expressed between the two conditions (due to somatic mutations or activities of its own upstream regulators), thereby affecting activities of its targets. Second, these targets might have somatic mutations in their regions that likely interact with the RBPs, which in turn affect their activities. We have included a remark about the somatic mutation in these RBPs themselves but refrained from dissecting what potential mechanisms beyond somatic mutation enrichment might be leading to differential activity. While the former setting is easier to detect (by direct differential expression analysis of RBP with tools such as DESeq), identifying the latter case first requires defining targets of the RBPs. SURF discovery module leverages the wealth of information SURF learns from shRNA-seq and eCLIP-seq data to define such direct targets for an RBP.*

*References*

*[1]	Vivian J, Rao AA, Nothaft FA, Ketchum C, Armstrong J, Novak A, et al. Toil enables reproducible, open source, big biomedical data analyses. Nature biotechnology. 2017;35(4):314– 316.*

*[2]	Goldman M, Craft B, Hastie M, Repeëka K, McDade F, Kamath A, et al.. The UCSC Xena platform for public and private cancer genomics data visualization and interpretation. Cold Spring Harbor Laboratory; 2019.*

*[3]	Mayr C, Bartel D. Widespread Shortening of 3’UTRs by Alternative Cleavage and Polyadeny-lation Activates Oncogenes in Cancer Cells. Cell. 2009 09;138:673–84.*

*[4]	Yuan F, Hankey W, Wagner E, Li W, Wang Q. Alternative polyadenylation of mRNA and its role in cancer. Genes & Diseases. 2019 10;.*

*[5]	Qian J, Hassanein M, Hoeksema MD, Harris BK, Zou Y, Chen H, et al. The RNA binding protein FXR1 is a new driver in the 3q26-29 amplicon and predicts poor prognosis in human cancers. Proceedings of the National Academy of Sciences. 2015;112(11):3469–3474.*

*[6]	Glasser E, Agrawal AA, Jenkins JL, Kielkopf CL. Cancer-associated mutations mapped*

*on high-resolution structures of the U2AF2 RNA recognition motifs.	Biochemistry.*

*2017;56(36):4757–4761.*

*[7]	Soneson C, Delorenzi M. A comparison of methods for differential expression analysis of RNA-seq data. BMC bioinformatics. 2013;14(1):91.*

*[8]	Xiong HY, Alipanahi B, Lee LJ, Bretschneider H, Merico D, Yuen RKC, et al. The human splic¬ing code reveals new insights into the genetic determinants of disease. Science. 2015;347(6218). Available from: https://science.sciencemag.org/content/347/6218/1254806.*

*[9]	Jaganathan K, Panagiotopoulou SK, McRae JF, Darbandi SF, Knowles D, Li YI, et al. Pre-dicting splicing from primary sequence with deep learning. Cell. 2019;176(3):535–548.*

*[10]	Bogard N, Linder J, Rosenberg AB, Seelig G. A Deep Neural Network for Predicting and Engineering Alternative Polyadenylation. Cell. 2019;178(1):91 – 106.e23. Available from: http://www.sciencedirect.com/science/article/pii/S0092867419304982.*

*[11]	Bailey TL, Boden M, Buske FA, Frith M, Grant CE, Clementi L, et al. MEME SUITE: tools for motif discovery and searching. Nucleic acids research. 2009;37(suppl 2):W202–W208.*

*[12]	Giudice G, Sánchez-Cabo F, Torroja C, Lara-Pezzi E. ATtRACT—a database of RNA-binding proteins and associated motifs. Database. 2016;2016.*

*[13]	Cienikova Z, Damberger FF, Hall J, Allain FHT, Maris C. Structural and mechanistic insights into poly (uridine) tract recognition by the hnRNP C RNA recognition motif. Journal of the American Chemical Society. 2014;136(41):14536–14544.*

*[14]	Dominguez D, Freese P, Alexis MS, Su A, Hochman M, Palden T, et al. Sequence, structure, and context preferences of human RNA binding proteins. Molecular cell. 2018;70(5):854–867.*

*[15]	Vejnar CE, Messih MA, Takacs CM, Yartseva V, Oikonomou P, Christiano R, et al. Genome wide analysis of 3’ UTR sequence elements and proteins regulating mRNA stability during maternal-to-zygotic transition in zebrafish. Genome Research. 2019;29(7):1100–1114.*

*[16]	Dember LM, Kim ND, Liu KQ, Anderson P. Individual RNA recognition motifs of TIA-1 and TIAR have different RNA binding specificities. Journal of Biological Chemistry. 1996;271(5):2783–2788.*

*[17]	Aznarez I, Barash Y, Shai O, He D, Zielenski J, Tsui LC, et al. A systematic analysis of intronic sequences downstream of 5’ splice sites reveals a widespread role for U-rich motifs and TIA1/TIAL1 proteins in alternative splicing regulation. Genome Research. 2008;18(8):1247– 1258. Available from: http://genome.cshlp.org/content/18/8/1247.abstract.*

*[18]	Wang I, Hennig J, Jagtap PKA, Sonntag M, Valcárcel J, Sattler M. Structure, dynam¬ics and RNA binding of the multi-domain splicing factor TIA-1. Nucleic acids research. 2014;42(9):5949–5966.*

*[19]	Legrand N, Dixon DA, Sobolewski C. AU-rich element-binding proteins in colorectal cancer. World journal of gastrointestinal oncology. 2019;11(2):71.*

*[20]	Yang EW, Bahn JH, Hsiao EYH, Tan BX, Sun Y, Fu T, et al. Allele-specific binding of RNA-binding proteins reveals functional genetic variants in the RNA. Nature communications. 2019;10(1):1–15.*

*[21]	Seiler M, Peng S, Agrawal AA, Palacino J, Teng T, Zhu P, et al. Somatic mutational landscape of splicing factor genes and their functional consequences across 33 cancer types. Cell reports. 2018;23(1):282–296.*

**Second round of review**

**Reviewer 1**

I appreciate that the authors have addressed my previous concerns, and I think the modified figures provide significant clarity and insight. I support publication as I think this work will be of significant interest to the RNA community

A couple minor comments:

1. For the (I believe newly added) de novo motif analysis (e.g. Sup. Fig. S35), I’m unclear what is the background used (or if there is one)? As the AAUAAA sequence matches the canonical cleavage and polyadenylation site motif, I wonder if this is not specific to TIA1 but rather might simply be a commonly enriched sequence in alternative polyA sites generally (as they have to have a AAUAAA-like sequence), unless other alternative polyA sites (that lack TIA1 enrichment) are used as a background for comparison.

2. I don’t believe Fig. 4b is referenced anywhere in the main text

**Reviewer 2**

None

**Authors Response**

**Point-by-point responses to the reviewers’ comments:**

Reviewer #1 Comment 1: “For the (I believe newly added) de novo motif analysis (e.g. Sup. Fig. S35), I'm unclear what is the background used (or if there is one)? As the AAUAAA sequence matches the canonical cleavage and polyadenylation site motif, I wonder if this is not specific to TIA1 but rather might simply be a commonly enriched sequence in alternative polyA sites generally (as they have to have an AAUAAA-like sequence), unless other alternative polyA sites (that lack TIA1 enrichment) are used as a background for comparison.”

*Response: We appreciate the reviewer #1 pointing out that “the AAUAAA motif matches the canonical cleavage and polyadenylation site motif.” For the de novo motif analysis, the background model was built from the input sequences since we aimed to investigate motifs associated with different types of ATR events at the RBP level. As a result, as the Reviewer #1 rightly pointed out, we should expect some specific sequences to be universally enriched for a given ATR type. In fact, a similar AAUAAA motif was also enriched in the APA class-associated sequence features of CPSF6-TAP and IGFBP2-IAP, among others. We view this as further evidence of biological relevance of SURF-identified location features and the motifs. We now remarked on the resemblance of this AAUAAA motif to the canonical cleavage and polyadenylation site motif and presented additional significant motifs for TIA1-TAP and TIA1-IAP (Additional file 1: Fig. S33). Both of these additional motifs are different from the previously identified TIA1 motif, i.e., polyU site, suggesting the motif multi-modality of TIA1 in different ATR event classes.*
